# Bioprinted Organoids: An Innovative Engine in Biomedicine

**DOI:** 10.1002/advs.202507317

**Published:** 2025-07-25

**Authors:** Zhengwei Li, Kai Li, Cheng Zhang, Yingying Zhao, Yiyuan Guo, Jia He, Shiyuan Chang, Xinyi Fang, Kaizheng Liu, Pingping Zhu, Zhenzhen Chen, Changshun Ruan

**Affiliations:** ^1^ State Key Laboratory of Metabolic Dysregulation & Prevention and Treatment of Esophageal Cancer, School of Life Sciences Zhengzhou University Henan 450001 China; ^2^ Tianjian Laboratory of Advanced Biomedical Sciences Zhengzhou 450001 China; ^3^ Research Center for Human Tissue and Organ Degeneration Institute of Biomedicine and Biotechnology Shenzhen Institutes of Advanced Technology Chinese Academy of Sciences Shenzhen 518055 China; ^4^ University of Chinese Academy of Sciences Beijing 100049 China

**Keywords:** adult stem cells, bioprinting, cancer cells, organoids, pluripotent stem cells

## Abstract

Bioprinted organoids integrate bioprinting technology with organoid research, enabling the simultaneous reconstruction of human tissue morphology and physiological function in vitro. This approach offers distinct advantages in organoid fabrication, particularly in terms of structural precision, tissue mimicry, and functional fidelity. By leveraging the complementary strengths of both technologies, bioprinted organoids allow for the fabrication of personalized, architecturally engineered models that more accurately replicate organogenesis, physiological processes, and disease progression. Herein, this review outlines the key advantages of bioprinted organoids, with a focus on their ability to precisely control morphology, dimensions, and spatial organization. Bioprinted organoids are further categorized into three types based on their cellular origins and summarize recent progress in their application for human tissue modeling. Finally, ongoing challenges and future possibilities are sketched out, offering insights for potential innovation and research directions in the field. Bioprinted organoids not only propel the advancement of organoid research but also drive the evolution of bioprinting technologies. This integrated approach represents a powerful synergy between biomanufacturing and clinical medicine to pave the way for a new era in biomedical science and personalized healthcare.

## Graphical abstract

Bioprinted organoids refer to either the printing of stem cells into tissue shape and subsequent differentiate into organoids, or assembling induced organoids as bioinks to replicate native organ. It enables the creation of miniaturized organs with complex architectures and physiological functions, potentially enhancing reproducibility, throughput, and functional maturity, serving as an innovative engine in biomedicine.

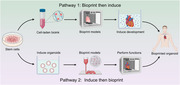

## 1 Introduction

The successful in vitro replication of human organs is crucial for the early identification of disease markers, the development of targeted therapies, and even the replacement of damaged tissues.^[^ ^1^ ^]^ Historically, researchers have relied on animal models to study the progression of diseases and evaluate corresponding therapeutic treatments. However, this approach has its inherent limitations, including interspecies variability, ethical concerns, and excessive time and costs.^[^ ^2^, ^3^ ^]^ Consequently, there is a growing urgency to construct models that closely mimic the structural and functional characteristics of human organs, while also allowing stable and long‐term culture.^[^ ^4^ ^]^ Advances in stem cell technology have enabled the utilization of pluripotent stem cells (iPSCs) and/or adult stem cells (ASCs) to form bioinspired mini‐tissues, known as organoids, which resemble natural tissues or organs.^[^ ^5^ ^]^ These conventional organoids emerge as self‐organized cellular constructs that developed within a controlled microenvironment in vitro, and exhibited partial structural and functional parallels to in vivo counterparts.^[^ ^6^ ^]^ As the field of organoid biology continues to advance, mini‐organs fabricated by bioprinting techniques, which have similar functions and structures to natural tissues, are also considered as the bioprinted organoids.^[^ ^7^ ^]^ Bioprinted organoids thus refer to either the printing of stem cells into tissue shape and subsequent differentiate into organoids, or assembling induced organoids as bioinks to replicate native organ. As an advanced extension of traditional organoid, the bioprinted organoids stand out for their ability to accurately control multi‐scale architecture, improve functional maturity, and address the structural heterogeneity and instability.^[^ ^8^ ^]^ Bioprinted organoids as a cornerstone in the burgeoning fields of precision medicine and regenerative medicine, as well as a promising avenue for drug screening and drug discovery.^[^ ^9^ ^]^

**Figure 1:**
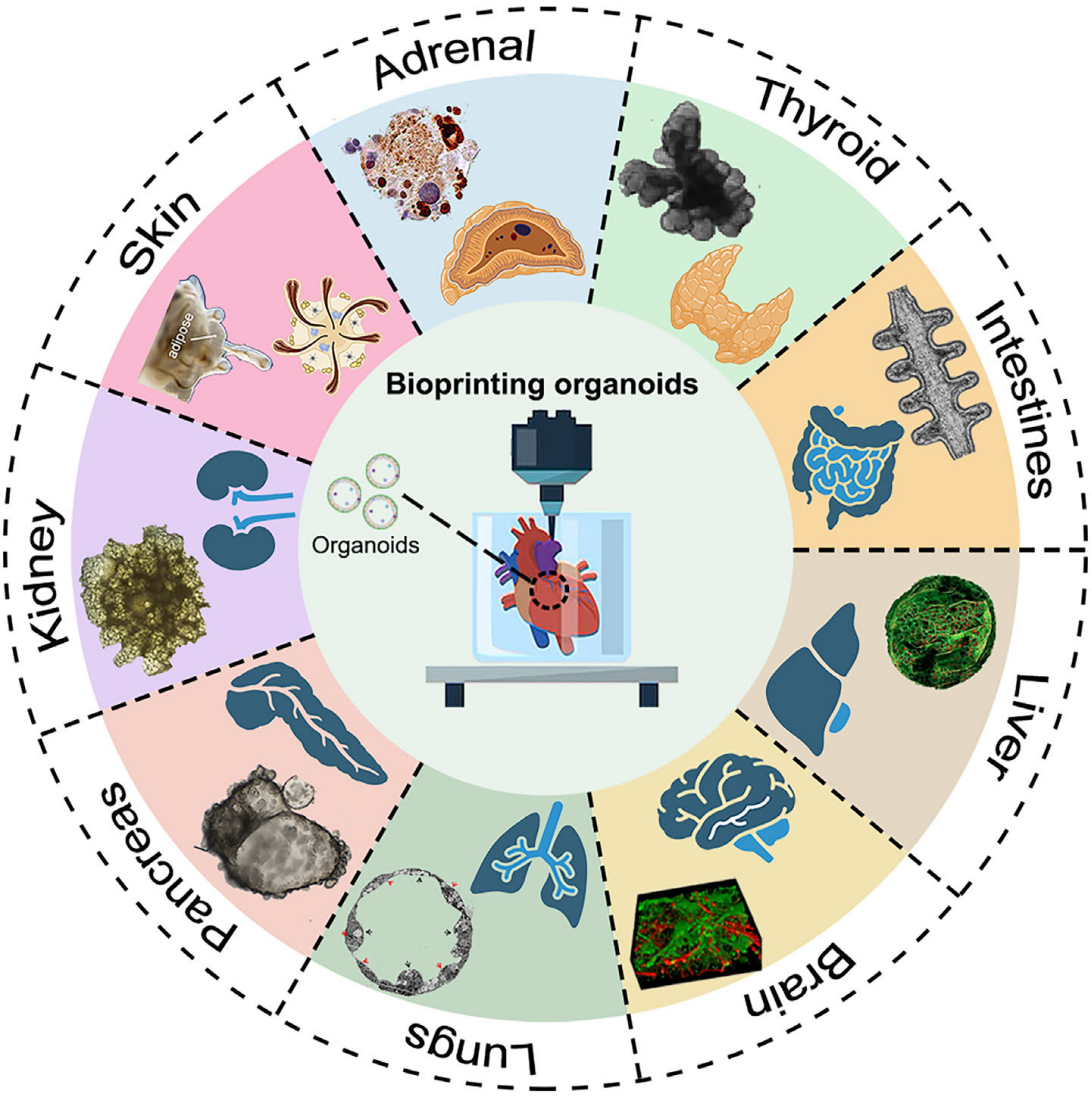
Schematic of a hybrid strategy integrating bioprinting and conventional organoid technology to merge structural control with self‐organization.^[^ ^13^, ^14^, ^15^, ^16^, ^17^, ^18^, ^19^, ^20^, ^21^ ^]^ Copyright 2023, 2021, 2019, Elsevier. Copyright 2020, 2020, 2020, 2013, 2020, Springer Nature. Copyright 2024, AAAS.

With the rapid evolution of bioprinting technology, research on bioprinted organoids has achieved significant milestones, heralding a future replete with boundless potential.^[^ ^10^ ^]^ The defining feature of bioprinting lies in its use of bioinks, which typically consist of cells, biomaterials, and growth factors. Bioprinted organoids involves the layer‐by‐layer printing of bioink according to a predefined structure, followed by in vitro culture and induction to form organoids with specific functions. Several bioprinted organoids techniques have been developed, including inkjet printing, extrusion‐based printing, laser‐assisted printing, and stereolithography‐based printing.^[^ ^11^ ^]^ Each of these approaches offers unique advantages such as high precision, scalability, and the ability to handle complex cellular arrangements. In contrast to conventional organoids, bioprinted organoids offer superior structural fidelity, enhanced functionality, personalized tailoring, improved production efficiency, and promising clinical potential.^[^ ^12^ ^]^ As shown in **Figure** **1**, a diverse array of conventional organoids representing various human systems have been developed, including these of the intestines, lungs, thyroid, adrenal glands, pancreas, skin, kidney, etc.^[^ ^13^, ^14^, ^15^, ^16^, ^17^, ^18^, ^19^, ^20^, ^21^ ^]^ By combining organoids with bioprinting technology, a hybrid strategy as bioprinted organoids has emerged, effectively bridging the gap between biological development and engineered structure.^[^ ^22^ ^]^ The goal is to harness the strengths of both methods, self‐organizing capabilities and spatial control, to overcome their individual limitations and drive in vitro organ reconstruction toward greater structural fidelity and physiological relevance. This groundbreaking technology showcases the versatility and far‐reaching implications of bioprinted organoids, demonstrating their capacity to revolutionize biomedical research, precision medicine and therapeutic innovation.^[^ ^23^ ^]^

As a rapidly advancing and critical area of biomidicial research, it is crucial to assess the current state of bioprinted organoid, address persisting challenges, and outline strategic directions for future development. This review begins by tracing the developmental trajectory of organoids, exploring diverse technologies, fabrication methodologies, and application models, with a particular emphasis on the unique advantages of bioprinted organoids. It then categorizes bioprinted organoids into three groups based on their cellular sources (iPSC, ASC, and cancer cells), highlighting their distinctive characteristics and potential applications. Finally, the review summarizes the key benefits and existing challenges of bioprinted organoids, while also forecasting promising breakthroughs and potential application horizons. The march of scientific and technological has derived us forward, so in vitro construction of human organs by bioprinted organoids becomes not just a possibility but a reality.

## 2 The Development and Advantages of Bioprinted Organoids

### 2.1 The Development Trajectory of Organoids

**Figure 2:**
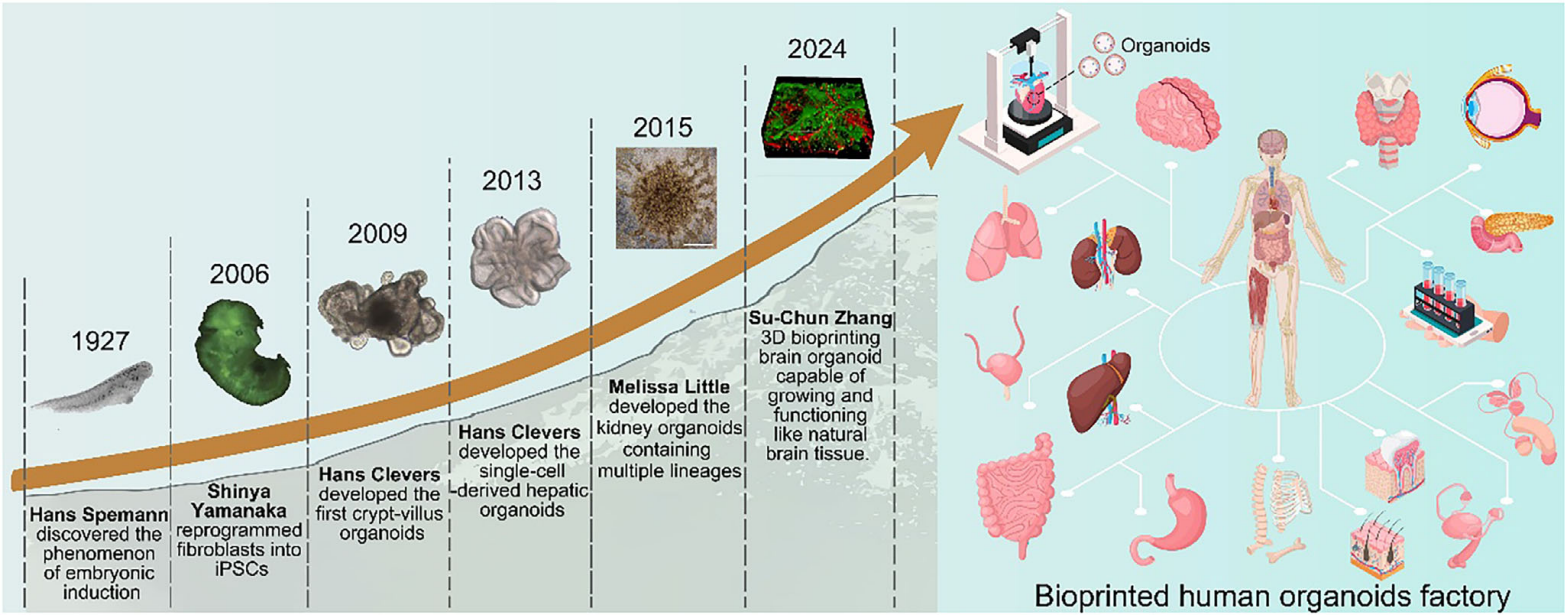
The milestone breakthroughs in the field of organoids since the 1900s and the future potential for bioprinted human organoids in the future.^[^ ^24^, ^26^, ^27^, ^30^, ^31^, ^32^ ^]^ Copyright 1907, AAAS. Copyright 2006, 2024, Elsevier. Copyright 2009, 2013, 2015, Springer Nature.

The origin of cell self‐organization can be traced back to 1910, when Professor H. V. Wilson discovered that mechanically separated sponge cells could regroup and self‐organize into new organisms.^[^ ^24^ ^]^ This discovery opened the door to in vitro tissue culture and laid the foundation for organoids, as shown in **Figure** **2**. Later, Tomohiro Kono achieved significant advancement by fusing different embryonic cells to form recombinant embryos that had the ability to develop in vitro.^[^ ^25^ ^]^ His research led to the birth of the first mouse produced by “parthenogenesis” through gene editing, which is an unprecedented achievement in history. These early investigations marked the initial stage of tissue development, mainly utilizing embryos or embryonic cells for in vitro tissue culture. To address these challenges, Shinya Yamanaka successfully cultivated iPSCs by reprogramming human skin cells and inserting four genes.^[^ ^26^ ^]^ These iPSCs have the potential to differentiate into various mature cells, such as fibroblasts, nerve cells, intestinal cells, and more, exhibiting similar functions to embryonic stem cells (ESCs). This breakthrough provided a solution to the long‐standing issue of cell sources for organoid research.

In 2009, Hans Clevers pioneered the cultivation of the first crypt‐villus organ using ASCs from mouse intestine, ushering a new era for organoids research.^[^ ^27^ ^]^ In 2013, organoids were named among the top ten scientific breakthroughs of the year by Science journal, and Clevers received the Scientific Breakthrough Award for his groundbreaking research in the field. Since then, researchers have successfully cultivated various organoid types, including kidneys, liver, lungs, intestines, brain, heart, pancreas, and more.^[^ ^28^, ^29^ ^]^

Hans Clevers et al. demonstrated that Lgr5 marks Wnt‐driven stem cell responsible for constitutive (e.g., intestinal, gastric) or intermittent (e.g., hair follicle) tissue self‐renewal. In a landmark study published in 2013, they further showed that a single Lgr5⁺ liver stem cell, when stimulated solely by Wnt signaling, could undergo unlimited expansion in vitro and form a self‐sustaining three‐dimensional organoid structure.^[^ ^30^ ^]^ These clonally derived liver organoids could be induced to differentiate in vitro and, upon transplantation into Fah⁻/⁻ mice, gave rise to functional hepatocytes. This breakthrough not only marked the transition of traditional organoid research from 2D cultures to fully three‐dimensional systems but also opened new avenues for exploring liver organoids in a wide range of biomedical applications. It remains one of the most representative and impactful studies in the field of liver organoid research to date.

Melissa and colleagues identified the spatiotemporal hierarchy between collecting duct progenitors and metanephric mesenchyme progenitors during kidney development. By using specific small molecules, they guided human pluripotent stem cells to self‐organize into complex, multicellular kidney organoids.^[^ ^31^ ^]^ These organoids recapitulate nephron structures, including proximal and distal and proximal tubules, early loops of Henle, and glomeruli containing podocytes that exhibit foot process formation and vascularization. Transcriptionally, they closely resemble the fetal human kidney. These kidney organoids represent powerful models for future biomedical applications such as nephrotoxicity screening, disease modeling, and serving as a potential source of therapeutic cells.

As bioprinting technology continues to advance and integrate with organoid research, a groundbreaking achievement was reported in February 2024 by Professor Su‐Chun Zhang and his team at the University of Wisconsin‐Madison.^[^ ^32^ ^]^ This innovative approach allowed neurons to form intricate connections both within individual layers and across multiple layers, ultimately creating a neural network structure that closely recapitulates the complexity of the human brain. This successful bioprinting of brain organoids marks a significant milestone in the field of bioprinted organoids. This achievement paves the way for the creation of increasingly complex, functional organoids, bringing the field closer to the ultimate goal of engineering patient‐specific neural tissues for research, drug testing, and therapeutic applications.

As biotechnology strides forward, bioprinted organoids will have a transformative leap in the future. It promises to extend a broader application scenarios, meticulously recreating diverse functions with remarkable precision and fidelity of human tissues and organs in vitro.^[^ ^33^ ^]^ Bioprinted organoids have propelled the field of biomedical research, made contributions to understand organ development, modeling diseases, and advanced regenerative medicine.

### 2.2 Advantages of Organoids Compared with Traditional Models

These conventional in vitro and in vivo biological models, such as 2D cell culture, cell spheroids, organ‐on‐a‐chip systems, patient‐derived tumor xenograft (PDX) mice, are widely employed to study signaling pathways, drug targets, and novel pharmacological effects. However, these models did not fully replicate human organ characteristics, which resulted in inconsistency of biological mechanisms.^[^ ^34^ ^]^ When using 2D cell culture models for research, the cell types are relatively homogeneous and even consisted of a single cell type. However, they are limited in their ability to accurately represent the in vivo microenvironment and immune system.^[^ ^35^ ^]^ Additionally, primary cells are often difficult to culture for extended periods and their genetic information became unstable during in vitro cultivation, which lead to significant differences in gene expression compared with in vivo conditions.^[^ ^36^ ^]^ Non‐human primate models are the most physiologically and immunologically similar to humans.^[^ ^37^ ^]^ Yet ethical constraints, exorbitant costs, complex and uncontrollable variables, and difficulty in conducting high‐throughput experiments restrict their broader application in biomedicine. PDX mice not only lack an immune microenvironment similar to that of humans, but also differ in brain development, metabolism, and other aspects.^[^ ^38^ ^]^ The success rate of experiments used PDX mice is difficult to guarantee due to the long experimental cycles and high costs.

Based on this, organoid technology emerged as the times require, which cultured from stem cells and exhibited physiological and biological characteristics that closely resemble of natural organs.^[^ ^39^ ^]^ At the same time, the development of gene editing technology allows cells to facilitate their rapid proliferation and in vitro culture when maintained genomic stability, which enables the expansion of various cell types to promote development of organoid technology.^[^ ^40^ ^]^ These organoids constructed by manipulated cells could fulfill requirements of personalized and high‐throughput testing, which addressed issues related to limited cell sources and insufficient quantities and granted advantage over traditional research models (**Table** **1**).^[^ ^41^ ^]^

**Table 1:** Advantages of bioprinted organoids compared with other traditional models.

Aspects	Bioprinted organoids	2D cell culture	Traditional organoids	Animal models	Organ‐on‐a‐chip
Structural bionics	**✓ High**: complex 3D structures	Low: single‐layer cells	Medium: simple structure	Low: determined structure	Medium: part of organ structure
Morphological plasticity	**✓ High**: Personalized design	Low: no 3D Morphology	Low: no 3D Morphology	Low: no plasticity	Medium: part of plasticity
Physiological similarity	**✓ High**: closer to the real human tissues	Low: lacks internal environment	Medium: simulates specific environment	**✓ High**: simulates internal environment	Medium: simulates specific environment
Cellular diversity	**✓ High**: Multiple cell types	Low: mainly single cell type	Medium: limited cell types	**✓ High**: natural cell types	Low: Two or more cell types
Disease model	**✓ Effective for pathological model**	Difficulty simulating conditions	Simulating pathological conditions	Accurate for certain diseases	Simulates diseases interactions
Drug‐screening	**✓ High**	Medium to high	Medium	Low	medium
Toxicology	**✓ High**: fewer species Differences	Low, hard to predicting	Medium: partial toxicology	**✓ High**: accurately predicting	Medium: partial toxicology
Ethical issues	**✓ Low**	**✓ Low**	**✓ Low**	Strict regulations	**✓ Low**
Preclinical progress	**✓ High**: Closer to human tissue	Low: limited research	Medium: species differences	**✓ High**: partial research	Medium: limited validation

Organoids faithfully reproduce intricate 3D architectures and microscale tissue interactions in vitro, thereby serving as physiologically relevant models of human organ systems.^[^ ^42^ ^]^ Especially in constructing cancer models and studying oncogenesis, cancer organoids provide a means to preserve the heterogeneity of tumor tissues.^[^ ^43^ ^]^ Furthermore, the gene expression patterns within cancer organoids show high consistency with in vivo conditions, thereby reducing genetic disparities between in vitro cultured and autologous tissue.^[^ ^44^ ^]^ Compared with traditional in vivo animal models for cancer research, cancer organoids exhibit shorter culture cycles, more stable cancer tissues, and can be utilized in high‐throughput experiments, effectively addressing the limitations associated with animal models.^[^ ^45^ ^]^

Organoids can enable personalized healthcare approaches that are challenging to achieve using traditional methods.^[^ ^46^ ^]^ Patient‐derived are used to construct organoids, ensuring a precise match with autologous tissue development and facilitating personalized drug screening.^[^ ^47^ ^]^ Additionally, the distinct variations and characteristics among individual patients can be taken into account to design unique treatment strategies and predict patient responses to treatment. From an ethical and legal perspective, the use of animal models has always been highly controversial. However, organoid technology offers a potential solution by reducing the reliance on animal experimentation.^[^ ^48^ ^]^ Organoids demonstrate higher predictive capabilities in terms of evaluating drug efficacy, side effects, and toxicity.^[^ ^49^ ^]^

Organoids offer significant advantages in mimicking the structure and function of human organs, while still face several pressing challenges that remain to be overcome. First, organoid culture requires precise environmental control, and maintaining stability and consistency remains a significant challenge, particularly in large‐scale cultures.^[^ ^50^ ^]^ Second, most organoids fail to effectively develop a vascular network in vitro. The lack of vascularization limits their size, functionality, and long‐term viability, which restricts their applications in drug screening and disease modeling.^[^ ^51^ ^]^ Moreover, organoids cannot fully recapitulate the complexity of organ functions, particularly in areas such as metabolism and immune responses, which remain inadequately reconstituted.^[^ ^52^ ^]^ Finally, organoids often exhibit limited precision, particularly in regulating variables such as cell distribution and cellular activity, which constrains their utility in disease research, toxicology assessments, and personalized medicine.^[^ ^53^ ^]^

In summary, organoids offer a more faithful representation of human physiological characteristics than traditional cell and animal models. Organoids provide a highly accurate and dependable platform for drug screening, demonstrate potential for personalized medicine, and reduce the need for animal experiments. These notable advantages highlight the significant potential of organoids for broad applications in medical research and clinical treatment.

### 2.3 Advantages of Bioprinted Organoids

In addition to traditional PSCs‐derived organoids, in vitro tissue constructs that mimic human organs functions can also be regarded as organoids in a broader sense.^[^ ^7^ ^]^ Bioprinting, due to its high precision, reproducibility, and customizability, is increasingly used to construct such extended forms of organoids. Different bioprinting techniques have unique advantages, as summarized in **Table** **2**. Each method has its own advantages and disadvantages, which makes them suitable for specific applications.^[^ ^54^ ^]^

**Table 2:** The advantages and disadvantages of different bioprinting techniques.

Bioprinting technology	Advantages	Disadvantages
Extrusion‐based bioprinting	High material compatibility Low cost Multi‐material support High cell density	Low resolution Cell damage Structural deformation
Inkjet bioprinting	High speed High resolution High cell viability Multi‐nozzle capability	Low viscosity limitation Nozzle clogging risk Post‐processing required
Laser‐assisted bioprinting	Nozzle‐free operation Single‐cell precision High‐viscosity compatibility	High equipment cost Dependence on materials
Digital light processing	Ultra‐high resolution No shear stress Smooth surface finish	Phototoxicity Limited material options High cost
Suspension support bioprinting	Soft hydrogel compatibility Anti‐collapse architecture	Support removal risks Limited commercial adoption

The earliest method employed in organoid research was in vivo tissue culture, which involved isolating human tissue for in vitro cultivation and conducting various biological, biochemical, and pharmacological experiments.^[^ ^55^ ^]^ This technology aided in understanding disease development and provided treatment approaches while minimizing impact on patients. Organ transplantation, commonly used in clinical medicine, can also be viewed as a form of living tissue culture, where the cultured material was transferred to the recipient's body.^[^ ^56^ ^]^ However, living tissue culture was complex and necessitated precise control of culture conditions, maintaining normal cellular and tissue physiology, and the provision of elaborate culture media and equipment.^[^ ^57^ ^]^ Replicating the intricate environment of the human body, such as mechanical forces, blood supply, and immune interactions, often posed challenges for living tissue culture. It is important to note that obtaining living tissue from the human body raises ethical concerns, which requires adherence to ethical guidelines and legal provisions.^[^ ^58^ ^]^

Apart from in vitro tissue culture, one of the most commonly and extensively used method was direct cell culture, in which stem cells were obtained and then cultured to proliferate and differentiate into desired organoids.^[^ ^59^ ^]^ This method allowed for precise control of cell growth, differentiation, and functional expression by manipulating the conditions and components of the culture medium.^[^ ^60^ ^]^ Using the patient's own cells for organoid reduced the reliance on exogenous materials, thereby minimizing the risk of immune rejection.^[^ ^61^ ^]^ Moreover, this method enabled standardized and scalable operations, improved repeatability, and held significant promise for clinical applications and drug screening. The source and purity of cells were crucial factors for organoid development, while challenges also existed in obtaining specific cell types and scaling up the culture process.^[^ ^62^ ^]^ While certain tissues and organs could be generated through direct cell culture, fully reproducing the structure and function of organs remained difficult.^[^ ^63^ ^]^

Organ‐on‐a‐chip technology integrates microfluidics, bioengineering, and cell biology to create miniature platforms that replicate key physiological functions of human organs in vitro. By precisely controlling parameters such as flow rate, shear stress, and nutrient delivery, these chips effectively simulate the mechanical and chemical cues found in native tissues, offering a powerful tool for investigating dynamic organ‐level processes.^[^ ^64^ ^]^ Organ‐on‐a‐chip can enhance research efficiency by enabling simultaneous simulation of multiple organ conditions, facilitating multiple types of experiments, expediting drug screening, and driving the development of disease models.^[^ ^65^ ^]^ Owing to their small scale and minimal sample requirements, organ‐on‐a‐chip systems are also well suited for medium‐to‐high‐throughput drug screening and toxicity evaluation.^[^ ^66^ ^]^ Due to technological limitations, organ‐on‐a‐chip typically only simulates certain functions and struggles to fully replicate the complex structures and functions of complete organs. Moreover, organ‐on‐a‐chip requires high‐quality cell, and challenges in large‐scale cultivation and maintenance further limit its application scope, causing its moderate efficiency.^[^ ^67^ ^]^ With automation and integration, organ‐on‐a‐chip technology can now process multiple organ models in parallel while incorporating big data analysis.^[^ ^68^ ^]^ Its performance in high‐throughput screening is approaching that of traditional cell culture methods and, in some areas, even exhibits higher biological relevance.

**Figure 3:**
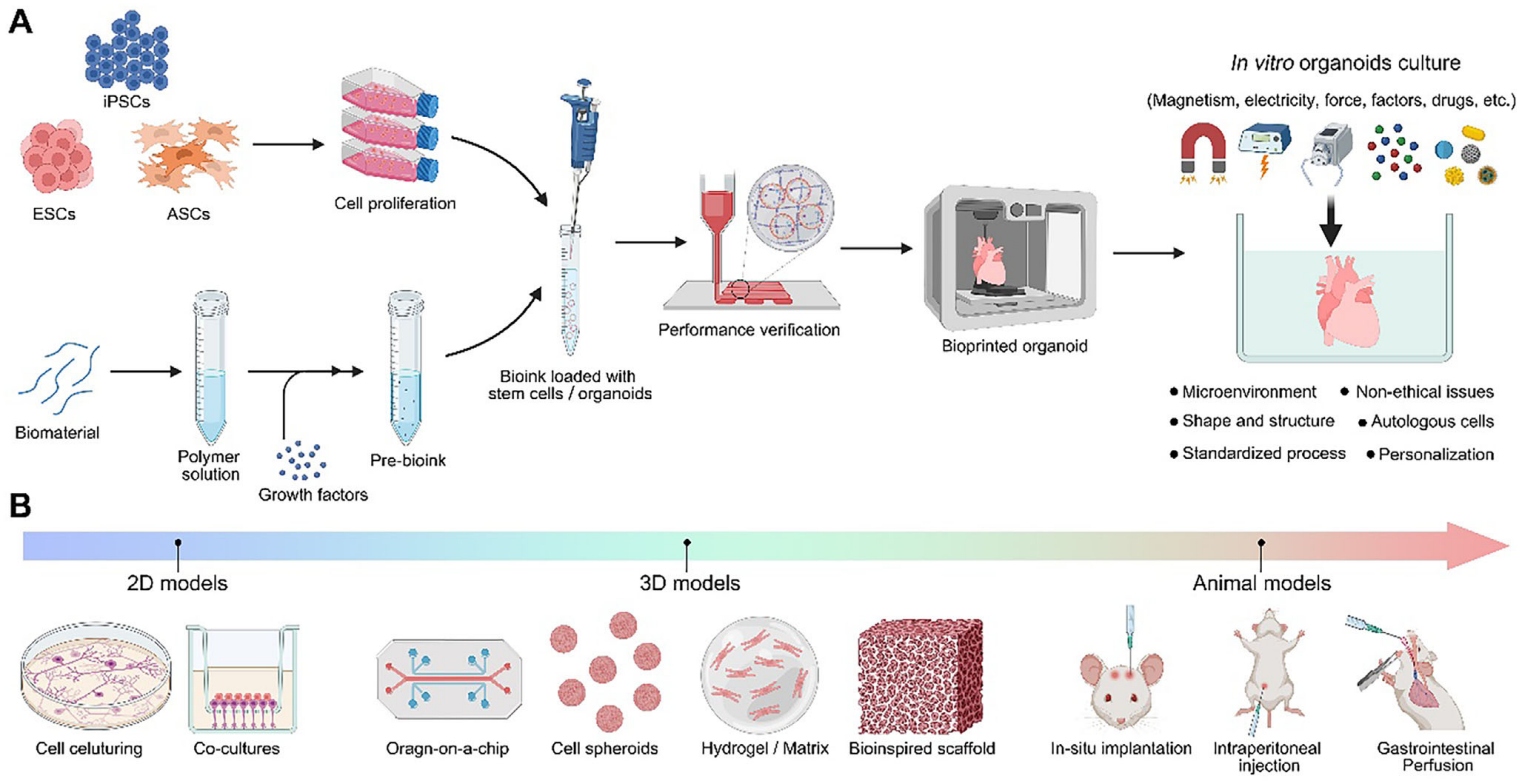
Advantages of bioprinted organoids compared with traditional models. a) Schematic diagram of the process for bioprinted organoids derived from human own cells and its advantages. b) Traditional biological models include 2D models, 3D models, and animal models. Figures form biorender.com.

Bioprinting, currently one of the most prominent research areas in material formation, plays an irreplaceable role in the construction of human tissues and organs in vitro.^[^ ^69^, ^70^ ^]^ By presetting ideal models, bioprinting allows accurate arrangement of cells, biomaterials, growth factors, and other components into 3D structures that resemble organs, enabling the personalized and bioinspired construction of human tissues and organs.^[^ ^71^, ^72^ ^]^ Compared with other methods, the main advantage of bioprinting lies in its precise control over the shape, size, and structure of models, making it possible to create fully personalized organs based on patients' specific needs and anatomical structures, as shown in **Figure** **3**.^[^ ^73^ ^]^ Moreover, bioprinting technology can use various biomaterials to achieve the bioinspired construction with complex structures and functions, which provides greater flexibility and adaptability in the fabrication of organoids.^[^ ^74^ ^]^ Bioprinted organoids offer an expanded scope of application across diverse settings and can function even under extreme weather or unique environmental conditions, a feat that eludes other methods.^[^ ^75^ ^]^ As an advanced 3D printing technology, bioprinting allows for precise control over the spatial distribution of cells and biomaterials at the micron level, enabling the creation of organoids with tailored shapes, functions, and biological properties.^[^ ^76^ ^]^ By simulating the natural arrangement and interactions of cells, bioprinting improves the biological functions and physiological characteristics of the organoid, which presents a promising alternative to traditional animal testing.^[^ ^77^, ^78^ ^]^

Bioprinted organoids have demonstrated numerous advantages over traditional organoids, but they still face various challenges. These challenges include the difficulty in maintaining the physiological properties of organoids over extended periods in vitro, the inability to recreate intricate vascular networks that closely resemble those of human tissues, a limited variety of bioinks available for printing, high development and application costs, and the absence of standardized quality control systems.^[^ ^79^ ^]^ However, as these issues are gradually addressed, bioprinted organoids are expected to become an indispensable tool in future medical practice, supporting more efficient, safe, and personalized treatments.

## 3 Bioprinted Organoids Derived from Pluripotent Stem Cells

Bioprinted organoids effectively preserved the morphology, viability, and differentiation capacity of stem cells, and closely resembled primary tissues. This resemblance enabled meticulous observation of biological processes, significantly enhancing research potential in the fields of human physiology and developmental biology.^[^ ^80^ ^]^ They have been extensively employed in personalized medicine, regenerative medicine, gene therapy, and transplant research.^[^ ^81^ ^]^ In this review, we delved into the progress of differentiation techniques in vitro and explored the distinctions among organoids derived from different types of stem cells. For organs that were challenging to obtain from human tissue, such as neuroectodermal organs, mesodermal renal organs, and endoderm‐derived epithelial organs, are typically cultured in vitro from pluripotent stem cells.^[^ ^82^ ^]^ By analyzing the challenges encountered in these applications, researchers can deepen their understanding of organoids derived from pluripotent stem cells and promote their broader use in both fundamental research and clinical treatment.

### 3.1 Organoids Derived from Embryonic Stem Cells

Although animal models have proven to be highly valuable for investigating development issues, fundamental interspecies differences have limited the applicability of insights from these models to human development.^[^ ^83^ ^]^ ESCs exhibit pluripotency and are able to differentiate into various cell types, including those found in different organs.^[^ ^84^ ^]^ This unique capability positions them as a promising resource for constructing diverse organoids, thereby advancing research and development in regenerative medicine.^[^ ^85^ ^]^ The differentiation scheme for bioprinted organoids derived from ESCs is based on three strategies: embryoid body formation, tissue induction, and subsequent maturation and differentiation.^[^ ^86^ ^]^ Since ASCs are often difficult to extract directly from natural tissues, ESCs offer a unique advantage for the successful bioprinting of various types of organoids. Moreover, the self‐renewal capacity and high uniformity of ESCs allow for long‐term in vitro cultivation without losing pluripotency, which is an important consideration for bioprinted organoids.

**Figure 4:**
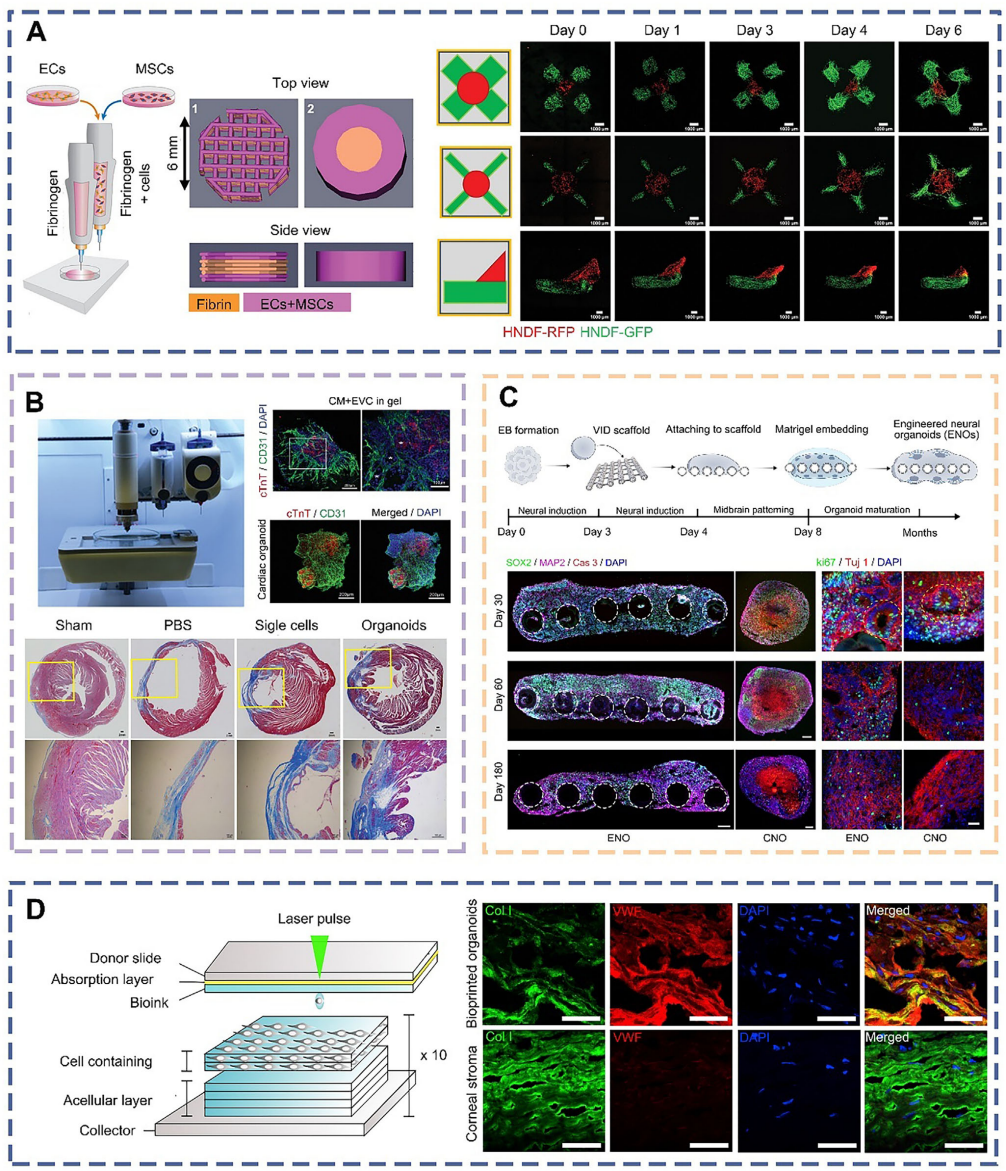
Schematic diagram of different bioprinted organoids derived from ESCs and their structural reconstruction in vitro. a) Schematic diagram of bioprinted pancreatic organoids and fluorescent images of cells in bioprinted organoids over time.^[^ ^87^ ^]^ Copyright 2024, Wiley. b) Schematic diagram of bioprinted cardiac organoids and representative images of Masson staining after implantation into ischemic regions.^[^ ^88^ ^]^ Copyright 2022, Wiley. c) Schematic diagram of bioprinted engineered neural organoids and representative immunostaining images of the ENOs and the CNOs. Scale bars, 200 µm.^[^ ^89^ ^]^ Copyright 2025, Elsevier. d) Schematic diagram of bioprinted corneal organoids and the stromal composition of 3D bioprinted organoids after 7 days. Scale bars, 1 mm.^[^ ^90^ ^]^ Copyright 2018, Elsevier.

Pancreas models offer significant potential for the development of therapeutic strategies for pancreatic disorders. However, existing models predominantly focus on insulin‐secreting β‐cells or pancreatic cancer cells, neglecting other essential components required for reconstructing the pancreas. Shulamit et al. created pancreatic organoids containing both endocrine and exocrine compartments by bioprinting mouse ESC‐derived pancreatic progenitors, pancreatic endothelial cells, and MSCs (**Figure** **4**a).^[^ ^87^ ^]^ The bioprinted organoids maintained their original structure by increasing the stiffness of the construct, without compromising control over cell positioning and shape preservation. The presence of the endothelial component in the bioprinted organoids significantly promoted endocrine differentiation while suppressing exocrine differentiation. These bioprinted organoids enabled the investigation of how structural design and cellular composition influence pancreatic cell fate, holding great promise for modeling pancreatic diseases and development, as well as for pharmaceutical testing.

In a quest to assess the efficacy of cell therapies for myocardial infarction, Liu et al. utilized early vascular cell spheres derived from ESCs as foundational seeds, combined with cardiomyocytes in a hydrogel matrix, to bioprint cardiac organoids replete with intricate microvasculature (Figure 4b).^[^ ^88^ ^]^ These bioprinted organoids exhibited a marked increase in the total number of junctions, vessel density, and relative sprout length, significantly outperforming single‐cell constructs in terms of vascularization. Upon implantation into the ischemic regions of a myocardial infarction mouse model, the bioprinted cardiac organoids led to a decrease in fibrotic area and an increase in left ventricular wall thickness, which contributed to improved cardiac function. This bioprinted cardiac organoids, derived from ESCs and featuring well‐organized, high‐density microvasculature, not only enhanced the viability of transplanted cells but also showed great promise in advancing cell therapies for myocardial infarction.

Guo et al. engineered brain organoids by integrating a bioprinted scaffold that mimics the physical and biological diffusion properties of human brain tissue with neural organoids derived from embryonic stem cells (Figure 4c).^[^ ^89^ ^]^ Experimental results showed that the bioprinted brain organoids significantly improved cell viability, reduced cellular stress, sustained neurogenesis, enhanced region‐specific differentiation, and exhibited more intense and complex neural activity. These findings indicate that bioprinted organoids can serve as scalable, reproducible, and cost‐effective models with functional properties, capable of mimicking pharmacological responses similar to those of human tissue, thereby offering new insights into clinical therapies and drug screening.

For individuals afflicted with corneal diseases, the poor long‐term success of corneal transplants is largely due to the lack of epithelial renewal, necessitating innovative approaches to improve function of corneal grafts. To this end, Heli et al. successfully fabricated corneal organoid using laser‐assisted bioprinted technology, employing limbal epithelial stem cells derived from ESCs to form the epithelial layer, and adipose stem cells to construct a stratified matrix (Figure 4d).^[^ ^90^ ^]^ These bioprinted organoid exhibited desirable epithelial cell morphology, expression of proliferation markers, and co‐expression of corneal progenitor markers, all of which contribute to a horizontally organized tissue structure akin to native cornea stroma. After 7 days in porcine organ cultures, the matrix of the bioprinted organoids integrated with the host tissue, accompanied by cellular migration from the organoid into the surrounding tissue. This research demonstrated that bioprinted organoids, with an intricate architecture resembling natural corneal tissue, substantiate the potential of ESCs as a resource for corneal grafts, offering new horizons for ocular surface reconstruction.

The emergence of organoids, resembling miniaturized organ‐like structures, provides an alternative and reliable avenue for studying development.^[^ ^91^ ^]^Apart from typical neural organs like the brain and retina, ECSs also play a crucial role in constructing various types of neural organoids, including hippocampal, thalamic, hypothalamic and midbrain, cerebellum organoid.^[^ ^92^ ^]^ In addition to the aforementioned organoids, ESCs also play a pivotal role in the formation of epithelial tissue organoids.^[^ ^93^ ^]^ By employing techniques such as bioprinting and directed differentiation, researchers have generated diverse organoids, further highlighting the significant contribution of ESCs in fundamental research. These organoid systems offer fundamental research tools for understanding the molecular mechanisms that govern cell fate specification and neural circuit assembly during developmental. Additionally, they hold great promise for drug discovery and for enhancing our understanding of the mechanisms underlying various diseases.

### 3.2 Organoids Derived from Induced Pluripotent Stem Cells

iPSC technology involves the transformation of mature adult cells into a pluripotent state, followed by their differentiation into specific cell lineages.^[^ ^94^ ^]^ This advancement has opened new avenues in regenerative medicine, disease modeling, and drug screening.^[^ ^26^ ^]^ iPSCs represent a distinct cellular source, sharing similarities with ESCs in terms of gene expression and their ability to develop into all three germ layers.^[^ ^95^ ^]^ This pluripotent state makes them valuable for modeling organ development and for various applications in organoid research.^[^ ^96^ ^]^ Human iPSCs are more sensitive to handling procedures, especially when dissociated into single cells, as their pluripotency and directed differentiation can be influenced by various environmental factors, including the culture medium, biomaterials, and cell density.^[^ ^97^ ^]^ To investigate the impact of bioprinting on cell behavior, pluripotency, and differentiation, researchers utilized undifferentiated iPSCs with biomaterials in a laser bioprinting system. They found that iPSCs were indeed more sensitive to the applied biomaterials used, but were not affected by the laser printing technology. This finding suggested that iPSCs could be successfully used to manufacture organoids via laser bioprinting without losing their pluripotency, provided that appropriate biomaterials are used. These bioprinted organoids derived from iPSCs have not only provided unprecedented opportunities for understanding the causes and pathophysiological mechanisms of various diseases, but have also yielded promising results in clinical trials aimed at treating degenerative diseases and repairing organ injuries.^[^ ^98^ ^]^

**Figure 5:**
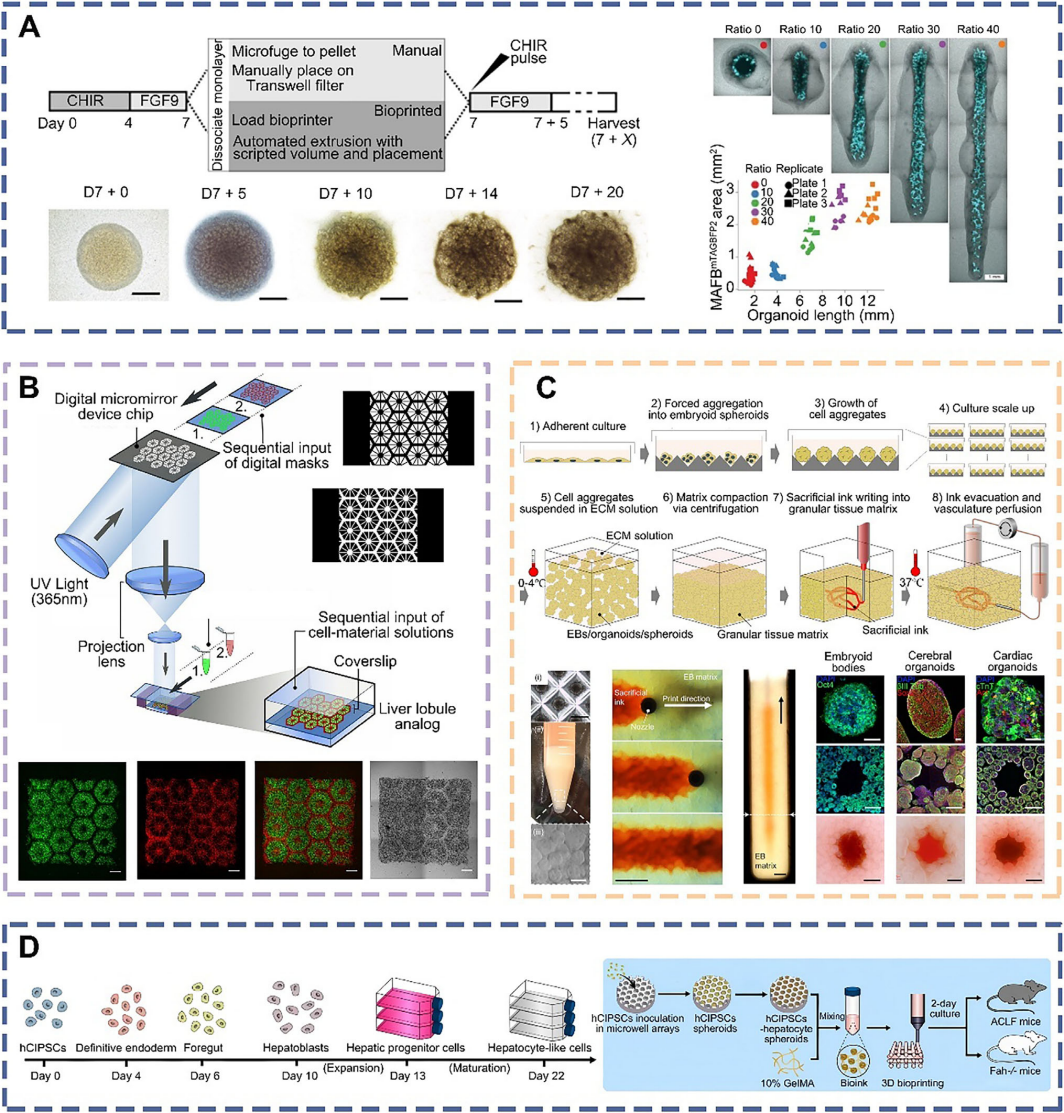
Schematic diagrams and functional verification of bioprinted organoids derived from iPSCs. a) Comparison of protocols for manually formed versus bioprinted kidney organoid formation and fluorescence imaging of live bioprinted organoids.^[^ ^18^ ^]^ Scale bars, 800 µm. Copyright 2020, Springer Nature. b) Schematic of a two‐step 3D bioprinting approach of hydrogel‐based hepatic organoids and fluorescence imaging on day 0. Scale bars, 500 µm.^[^ ^99^ ^]^ Copyright 2016, PNAS. c) Schematic of the SWIFT process showing various bioprinted organoids produced via embedded 3D printing within an EB matrix, observed from beneath the reservoir^[^ ^100^ ^]^ Copyright 2019, AAAS. d) Schematic illustrating the cell differentiation process, experimental workflow and bioprinted hepatocyte organoids used to treat two mouse models of liver failure in mice^[^ ^101^ ^]^ Copyright 2025, Wiley.

Melissa et al. utilized an extrusion‐based 3D bioprinting method to efficiently produce kidney organoids driven from iPSCs, which displayed a consistent pattern of functional proximal tubular segments and highly reproducible cell counts and vitality levels (**Figure** **5**a).^[^ ^18^ ^]^ The 3D bioprinting allowed precise manipulation of the physical and cellular properties of these kidney organoids, including their size, cell count, and configuration. The bioprinted organoids derived from iPSC led to improvements in production yield, quality control, scalability, and structural integrity, which have enhanced their feasibility for applications involving human kidney tissues both in vitro and in vivo.^[^ ^102^ ^]^ This study also holds significant promise for applications in drug screening, disease modeling, and the development of kidney substitutes.

Chen et al. developed various bioinks by combining hepatic progenitor cells derived from iPSCs, human umbilical vein endothelial cells, and adipose stem cells in a hydrogel matrix, and then bioprinted liver organoids with micro‐scale hexagonal structures (Figure 5b).^[^ ^99^ ^]^ This approach improved the morphological organization of hepatic progenitor cells within bioprinted organoids, resulting in elevated liver‐specific gene expression, increased secretion of metabolic products, and enhanced cytochrome P450 induction. This innovative study enhanced the structure and function of bioprinted liver organoids derived from iPSCs, which showed significant potential for early‐stage personalized drug screening and in vitro studies of hepatic pathophysiology.

Lewis et al. employed a bioprinting strategy that relies on sacrificial hydrogels to generate functional tissues composed of bioactive matrices, leading to organoids with high cell density, advanced maturity, and desirable functionality (Figure 5c).^[^ ^100^ ^]^ Experimental results showed that bioprinted organoids perfused with normoxic or hyperoxic media exhibited similar cell viability. Meanwhile, endothelial cells formed characteristic fusion patterns on certain parts of the luminal surface, demonstrating vascular regeneration within the organoids. Further studies revealed that the cardiac organoids, bioprinted using this perfusable strategy, were able to spontaneously synchronize their contractions after 7 days of culture, exhibiting rhythmic and rapid calcium wave propagation. This approach, which utilizes bioactive matrices to bioprint personalized organoids with embedded vascular channels, exhibits biological functions that closely resemble those of human tissues, opening up new avenues for clinical therapies and drug development.

Pang et al. differentiated and expanded human chemically induced pluripotent stem cells (hCiPSCs) into hepatic progenitor cells through multiple stages, which were then matured in situ within microwells to generate hCiPSC‐derived hepatic organoids (Figure 5d).^[^ ^101^ ^]^ These organoids were subsequently mixed with GelMA and bioprinted via 3D bioprinting technology for the treatment of two different mouse models of liver failure. Experimental results demonstrated that the spheroid‐based bioprinting technique enabled high cell density, rapid spheroid formation, and rich intercellular interactions within the constructed liver organoids, showing significant therapeutic effects in treating liver failure. This study indicates that bioprinted liver organoids exhibit cell viability comparable to natural tissue and maintain liver‐specific functions, highlighting their significant potential for clinical research in regenerative medicine.

In traditional culture models, it was challenging for organoids derived from iPSCs to mature into fully developed tissues. Typically, these organoids resembled fetal tissues, which was attributed to two possible reasons: first, achieving tissue maturity required prolonged culture under conditions that were practically limited by current cultivation capabilities; second, the limited ability to fully replicate embryonic development led to the loss of crucial interactions between organoid cells and co‐developing cells.^[^ ^103^ ^]^ The combination of 3D bioprinting and iPSC technology holds promise for addressing the above issue, which has significant implications for regenerative medicine research. However, several obstacles remain, such as establishing xeno‐free and footprint‐free clinical‐grade iPSC reprogramming protocols, selecting suitable bioinks, and creating integrated bioreactor systems for tissue maturation.^[^ ^104^ ^]^

## 4 Bioprinted Organoids Derived from Adult Stem Cells

ASCs also possess the ability to differentiate into specific lineages, making them a promising cell source for bioprinted organoids and facilitating the study of critical aspects of tissue development.^[^ ^105^ ^]^ One notable advantages of ASCs is that they could be obtained from adult tissues without raising ethical concerns.^[^ ^106^ ^]^ Furthermore, ASCs, mostly sourced from the patient's or donor's body, are relatively easy to obtain, eliminating the need for preparing and maintaining complex cell production lines. iPSCs are generally considered to have lower immunogenicity, but the potential for immune reactions to allogeneic grafts cannot be ruled o ut. From this perspective, ASCs have emerged as a more favorable cell type for cell therapy and tissue engineering.^[^ ^107^ ^]^ In some cases, the boundary between stem cells and progenitor cells has not been clearly established. Therefore, this review treats ASCs and progenitor cells as similar cell types, and summarizes their roles in bioprinted organoid construction and future development prospects.

### 4.1 Organoids Derived from Mesenchymal Stem Cells

Mesenchymal stem cells (MSCs) possess a remarkable capacity for differentiation into multiple under suitable conditions, giving rise to diverse cell types such as osteocytes, adipocytes, chondrocytes, and neurons.^[^ ^108^ ^]^ As a result, MSCs can serve as seed cells, providing a robust cellular reservoir for constructing bioprinted organoids with specialized functions and complex architectures^[^ ^109^ ^]^ Moreover, MSCs exhibit immuneregulatory and anti‐inflammatory properties, which help reduce immune rejection and inflammatory responses during transplantation. These effects collectively enhance the survival and functionality of implanted organoids.^[^ ^110^ ^]^ Consequently, MSCs hold immense potential in the realm of bioprinted organoids for tissue engineering and regenerative medicine, providing a powerful platform for research and a promising therapeutic paradigm.^[^ ^111^ ^]^

**Figure 6:**
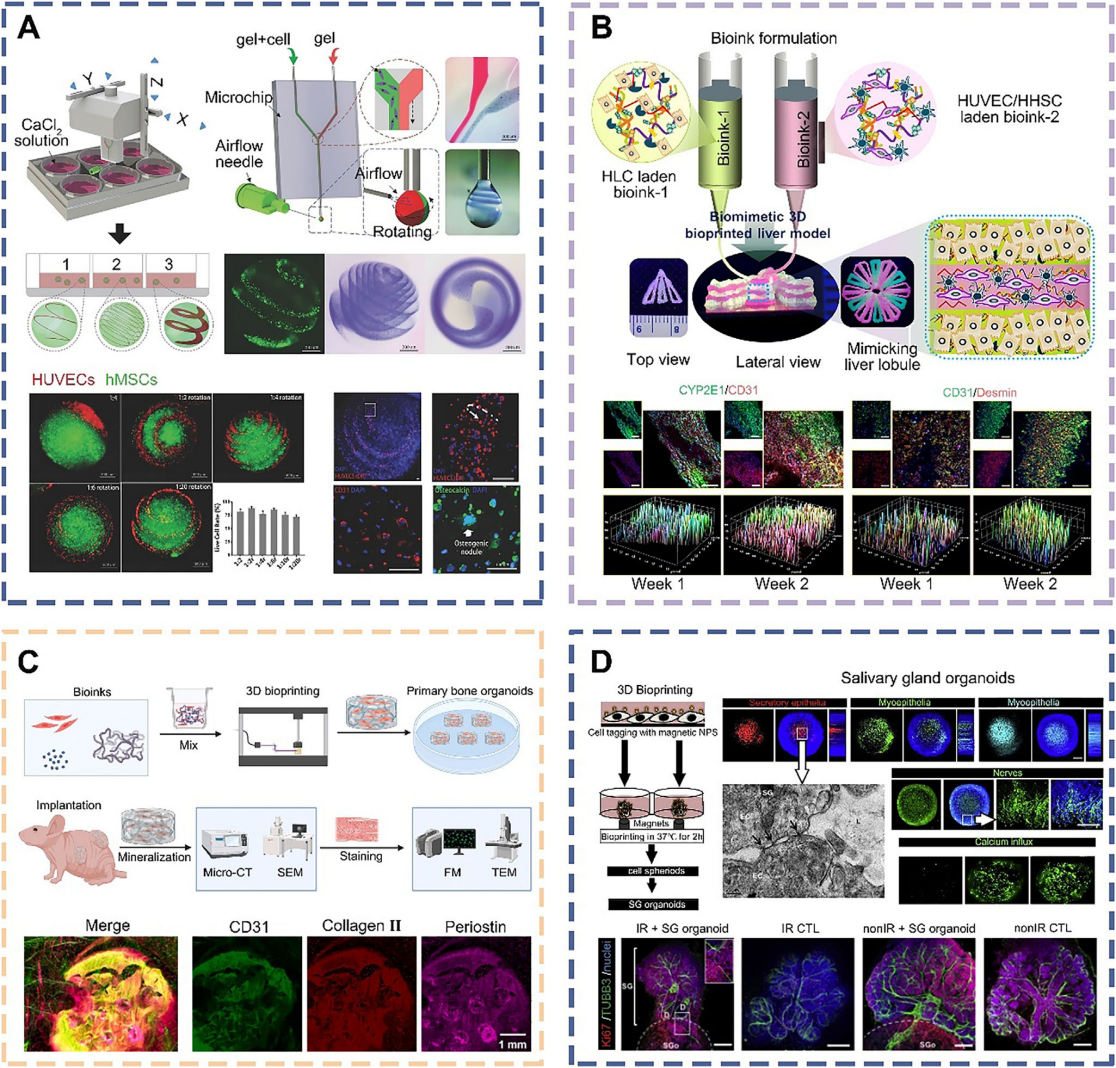
Schematic diagrams and applications of bioprinted organoids derived from MSCs. a) Schematic diagram of bioprinting 3D spiral‐based cell‐laden spheroids using airflow‐driven rotation to realize osteogenesis and angiogenesis.^[^ ^112^ ^]^ Copyright 2018, Wiley. b) The 3D liver model and bioprinted multilayered liver organoid are designed for use in high‐throughput drug screening platforms.^[^ ^114^ ^]^ Copyright 2022, ACS. c) 3D bioprinting of bone organoids along with their cultivation and in vivo assessment.^[^ ^115^ ^]^ Copyright 2025, Elsevier. d) Differentiation of cell spheroids into SG organoids for transplantation purposes using 3D bioprinting platforms.^[^ ^116^ ^]^ Scale bar, 200 µm. Copyright 2018, Elsevier.

He et al. developed a new airflow‐assisted bioprinting method to print multi‐functional spiral microstructures, such as spherical spirals, roses, and saddles (**Figure** **6**a).^[^ ^112^ ^]^ This microfluidic nozzle utilized laminar flow to accurately extrude an MSC‐laden alginate solution into several jets with different patterns and boundaries, which improved the ability to encapsulate complex cells and reconstruct spiral vascularized multi‐cellular organoids. This technology provided a precisely controlled spiral microstructure for multi‐cellular asymmetric microspheres with excellent resolution, serving as a powerful tool for manufacturing miniature organoids. In addition, embedding multiple cells in a sphere enabled the construction of functional organoids in vitro, which could provide various biomimetic asymmetric prototypes for basic medical research and regenerative medicine. Apart from airflow assistance, He et al. also developed an electrically assisted bioprinting method that generated low‐concentration methacrylated gelatin (GelMA) microdroplets loaded with MSCs.^[^ ^113^ ^]^ This method exhibited the advantages of low cost, low cytotoxicity, and high efficiency, making it a promising tool for constructing microdroplets in cell therapy, drug delivery, and organoid construction.

Biman et al. successfully combined hepatocyte‐like cells derived from human MSCs with silk fibroin hydrogel to create bioink, and then incorporated HUVECs and hepatic stellate cells with hydrogel as supportive materials (Figure 6b).^[^ ^114^ ^]^ Through in vitro bioprinting techniques, they engineered a liver organoid that faithfully mimicked the spatial arrangement and cellular composition of the natural liver. This bioprinted liver organoid featured a native‐like alternating structure with functional sinusoidal‐like luminal networks in both horizontal and vertical orientations, and exhibited enhanced albumin production, urea synthesis, and cytochrome P450 activity. Assessments of cell viability and metabolic function revealed that the organoid exhibited clinically relevant hepatotoxic responses to non‐hepatotoxic substances, specific hepatotoxic drugs, and hepatotoxic constituents, in a dose‐dependent manner, thus providing a robust platform for hepatotoxicity screening.

Su et al. mixed BMSCs with hydrogel to create a biomimetic bone matrix bioink and then employed photocuring‐based 3D bioprinting technology to fabricate bone organoids in vitro (Figure 6c).^[^ ^115^ ^]^ Upon subcutaneous implantation into nude mice, the bioprinted bone organoids were observed to spontaneously develop mineralized bone regions. With long‐term cultivation, these bioprinted organoids gradually matured into differentiated bone tissue, undergoing both mineralization and vascularization processes. This approach provides a novel platform for studying bone development and treating bone injuries through photocurable bioprinting.

Ferreira et al. utilized neural crest‐derived MSCs as seed cells and labeled them with magnetic nanoparticles. Using magnetic 3D bioprinting, they precisely manipulated magnetic field gradients to generate cellular spheroids. Upon stimulation with fibroblast growth factor, these spheroids developed into innervated salivary gland (SG) organoids (Figure 6d).^[^ ^116^ ^]^ When transplanted into an in vitro model, the SG organoids expressed key epithelial features of the salivary gland, including secretory epithelium, ducts, myoepithelial cells, and neurons. Under stimulation with various neurotransmitters, the organoids exhibited intracellular calcium mobilization and changes in transepithelial electrical resistance. These findings demonstrate that the bioprinted SG organoids possess functional innervation and biological activity, marking a critical step toward the in vitro regeneration of salivary glands and the development of therapies for xerostomia.

MSCs exhibit the ability to differentiate into various cell types, including osteocytes, chondrocytes, and adipocytes, which are extensively utilized and highly relevant to organoid construction.^[^ ^117^ ^]^ Additionally, MSCs are relatively abundant and can be easily isolated from diverse tissues, such as bone marrow, adipose tissue, and placenta, facilitating their procurement.^[^ ^118^ ^]^ However, compared to iPSCs, MSCs have more limited differentiation potential, which constrains their application in a wide range of treatments. Furthermore, meticulous culture conditions and stringent quality control measures are imperative during in vitro culturing, as they help preserve their pluripotent characteristics and ensure high differentiation potency.

### 4.2 Organoids Derived from Neural Stem Cells

Neurological disorders such as schizophrenia, bipolar disorder, Parkinson's disease, Alzheimer's disease, and epilepsy have plagued humanity for centuries.^[^ ^119^ ^]^ However, a comprehensive understanding of the causes and treatment methods for these diseases has remained elusive, primarily due to limitations in in situ brain research techniques. Neural organoids, derived from self‐organizing populations of stem cells through a “top‐down” bioprinting approach, represent a cutting‐edge field in the study of the mechanisms behind neurological diseases and potential treatments.^[^ ^120^ ^]^ Research on neural organoids not only contributes to the understanding of neurons communication and self‐organization, enabling higher‐resolution measurements of neural activity, but also provides valuable insights into the pathophysiology of neurological disorders.^[^ ^121^ ^]^

**Figure 7:**
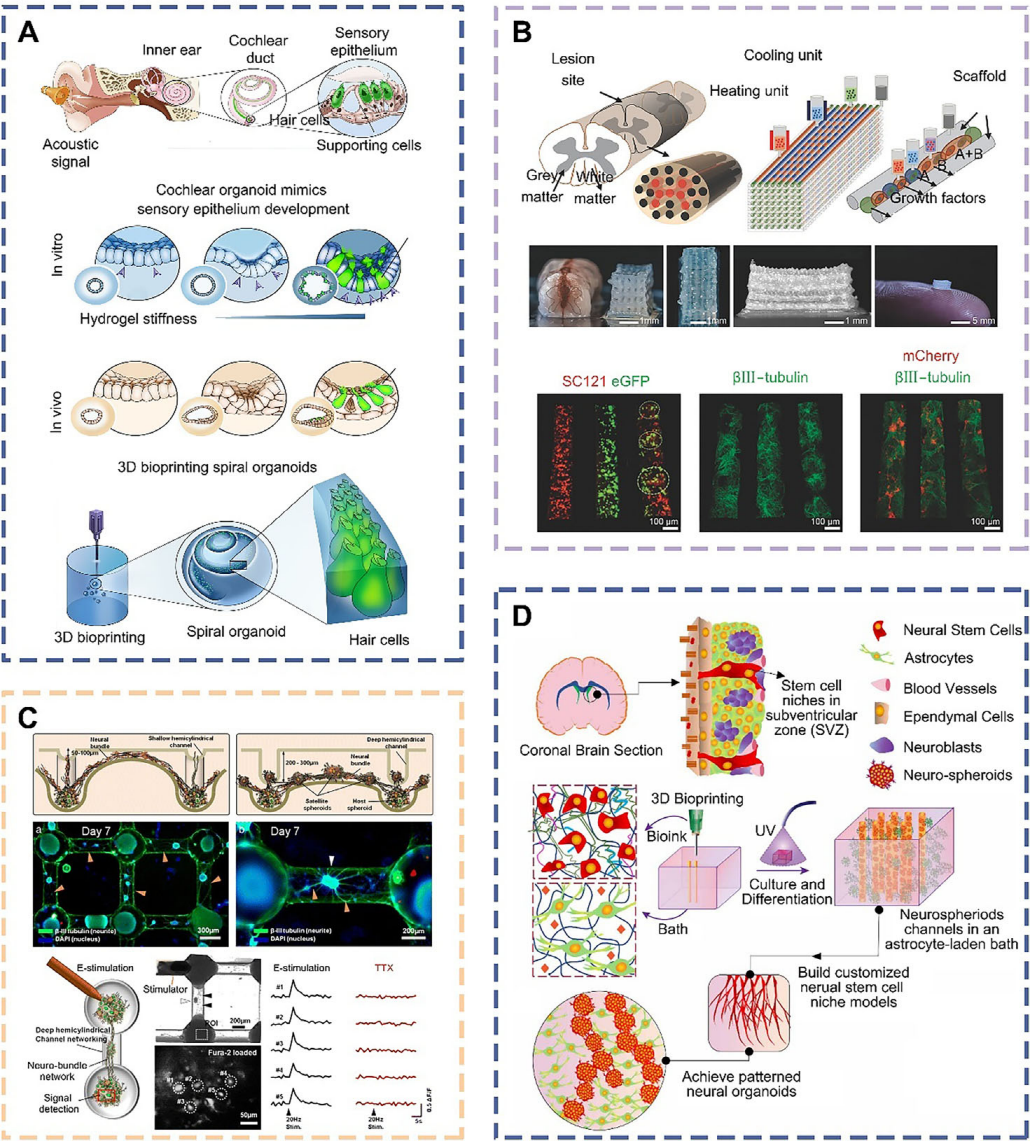
Schematic diagrams and applications of bioprinted organoids derived from NSCs. a) Schematic representation of hair cells in the sensory epithelium of the inner ear for acoustic perception, and bioprinted spiral cochlear organoids.^[^ ^122^ ^]^ Copyright 2023, AAAS. b) Schematic diagrams of a bioprinted spinal cord organoid, showing the distribution and functional expression of cell types in specific channels.^[^ ^123^ ^]^ Copyright 2018, Wiley. c) Schematic diagram and functional characterization of bioprinted organoids derived from neural stem cells.^[^ ^124^ ^]^ Copyright 2015, Springer Nature. d) Schematic diagram of bioprinted neural stem cell niches used to form coronal brain organoids.^[^ ^125^ ^]^ Copyright 2021, IOP.

Li et al. used highly biocompatible and mechanically controllable hydrogel bioink to bioprint spiral structures loaded with cochlear progenitor cells, thereby achieving the biomimetic construction of cochlear organoids in vitro (**Figure** **7**a).^[^ ^122^ ^]^ The organoid formation process revealed that the extracellular matrix (ECM) drove the development of sensory epithelium in a stage‐dependent manner through changes in matrix stiffness. The spiral cochlear organoids demonstrated high vitality and contained cells resembling hair cells. Additionally, moderate matrix hardness could stimulate the expansion of epithelial organoids. The current limitation of these cochlear organoids was a lack of close connections within the epithelial tissue and its microenvironment, hindering interactions among different cell types. Therefore, a crucial future task is to build networks for cell–cell communication to achieve vascularization and neuralization of organoids, thereby improving the replication of physiological characteristics and enhancing functional performance.

Structurally, spinal cord tissue is inherently heterogeneous, with various types of cells arranged in a complex spatial distribution. In pursuit of an optimal cellular spatial arrangement, Michael et al. bioprinted spinal cord organoids by clustering spinal cord neuron progenitor cells (sNPCs) and oligodendrocyte progenitor cells (Figure 7b).^[^ ^123^ ^]^ This innovative organoid features channels measuring 150 µm in width, spaced at 200 µm intervals, and exhibits precise cell alignment. The sNPCs underwent differentiation and axonal extension throughout the channels in the organoids, sustaining neural network activity. These organoids could serve as in vitro models that faithfully replicated the intricate organizational structure of the central nervous system. This organoid plays a pivotal role in studying complex central nervous system disorders, including spinal cord injuries, and holds potential as an implant for clinical treatments.

Lee et al. developed a concave hemispherical array scaffold with an integrated microchannel network using bioprinting technology. Within these deep hemispherical microchannels, different neural stem cells self‐assembled into spheroids, which served as anchoring points to facilitate interactions between adjacent spheroids, ultimately forming neural organoids (Figure 7c).^[^ ^124^ ^]^ During organoid formation, neural progenitor cells differentiated into both glial cells and neurons. Meanwhile, calcium ion signaling was monitored using imaging techniques, and it was observed that neurochemical signals induced by electrical stimulation could be transmitted across different regions of the organoid. These findings suggest that organoids constructed from bioprinted neural stem cells can replicate certain aspects of signal transmission in the human brain, making them a valuable in vitro model for studying brain function.

To address the challenge of precisely controlling the spatial distribution and composition of traditional organoids, Shin et al. employed embedded 3D bioprinting technology to fabricate structured organoids from neural stem cell spheroids (Figure 7d).^[^ ^125^ ^]^ These bioprinted neural organoids enabled spatially specific patterning and contained both neurotrophic factors and glial cells. They successfully recreated the neural stem cell niche and induced differentiation into neurons. This bioprinted neural organoid model mimics human neural development in vitro, thus providing a valuable experimental platform for neurological disease modeling, neural regeneration, and drug discovery and repurposing.

Through bioprinting technology, the arrangement of NSCs within a 3D structure to mimic heterogeneous cell distribution in the brain was accomplished, offering a substantial advantage over limited capabilities of traditional cell culture processes, which could only achieve restricted exploration through alternative or intersecting patterns.^[^ ^126^ ^]^ Additionally, the management of bioink aided in fostering the growth and development of NSCs within organoids, showcasing impressive neurite outgrowth and the establishment of functional neural networks. Furthermore, the incorporation of differentiation factors, along with the intricate design of tissue microstructures and controlled release of growth factors, further directed the migration and axonal extension of NSCs, resulting in the targeted differentiation of organoids. Although the bioprinted neural organoids were still in the early stages, they have already demonstrated substantial potential for reproducing complex neural tissues and microstructures in vitro.

### 4.3 Organoids Derived from Intestinal Stem Cells

Intestinal stem cells (ISCs) are located near the base of crypts, where they rapidly divided and transported proliferating daughter cells upward to occupy the remaining portion, migrating toward the sides of the villi.^[^ ^127^ ^]^ The most prevalent in vitro model employed for investigating intestinal barrier function or simulating drug absorption is the human intestinal epithelial cell line cultured on transwell inserts. However, this model fell short in depicting the spatial distribution of ISCs and tissue morphology, as well as in reconstructing other vital differentiation functions in the intestine.^[^ ^128^ ^]^ Bioprinting offers the capability to fabricate organoids featuring villus‐like structures and evenly distributed pores. These organoids presented an external muscular layer reminiscent of the inner layer of the intestinal epithelium, along with essential constituents of the intestinal stroma, including muscles, blood vessels, and nerves, closely resembling the natural intestinal structure.^[^ ^129^ ^]^ Consequently, bioprinted intestinal organoids and effectively inducing intestinal morphogenesis emerges as an ideal approach for addressing this issue.

**Figure 8:**
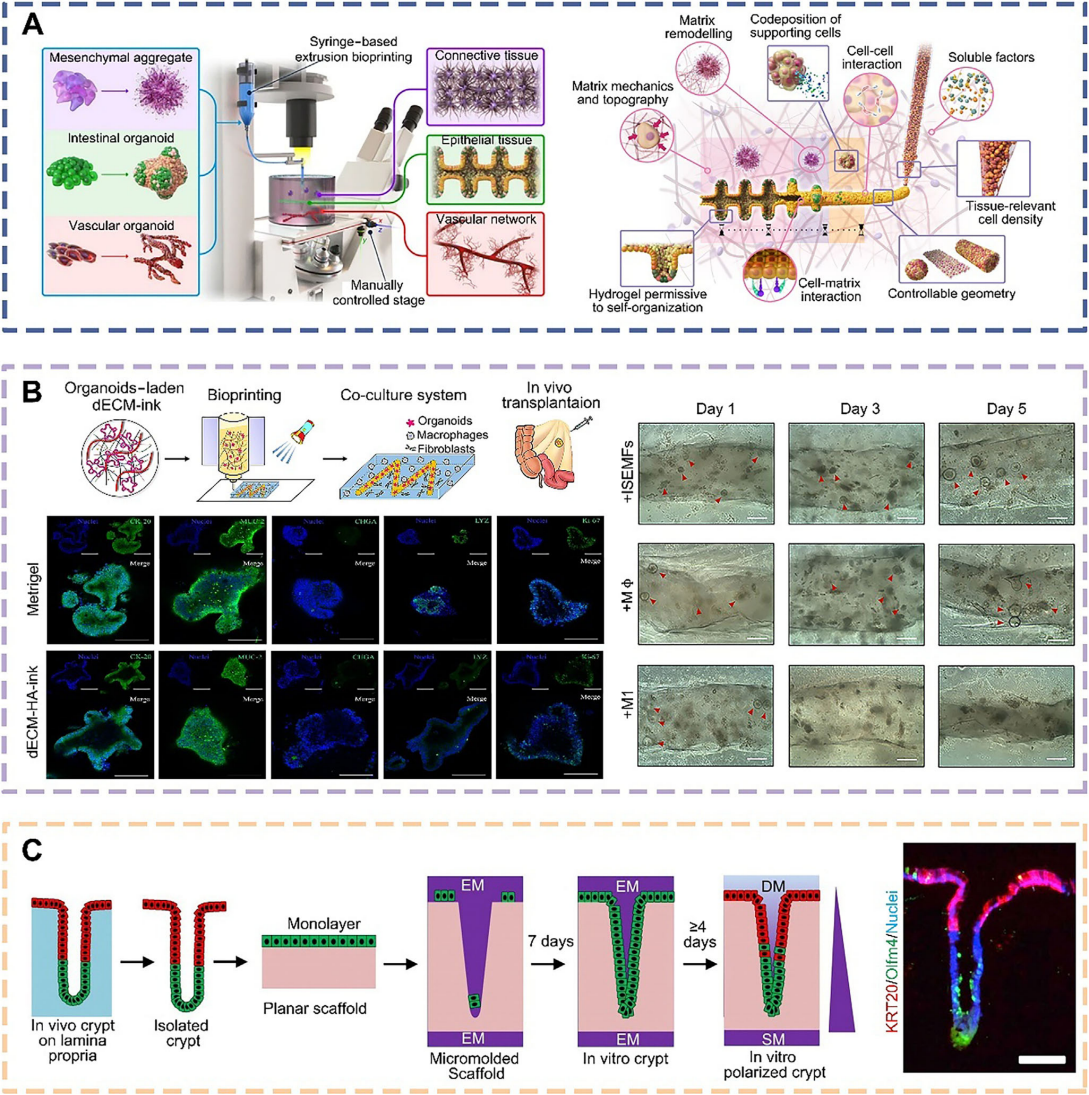
Schematic diagrams and applications of bioprinted organoids derived from ISCs. a) Schematic diagrams of a bioprinting‐assisted organoid fabrication strategy and bright‐field images of bioprinted organoids. Scale bars, 200 µm.^[^ ^130^ ^]^ Copyright 2020, Springer Nature. b) Schematic diagrams of fabricating bioink for bioprinting intestinal organoids and bioprinted intestinal tissues in bioink. Scale bars, 50 µm.^[^ ^131^ ^]^ Copyright 2023, Wiley. c) Schematic diagrams illustrating the fabrication of visible light‐activated colon dECM‐based bioink and the bioprinted tubular intestinal organoids.^[^ ^132^ ^]^ Copyright 2018, Elsevier.

Matthias et al. employed a bioprinting strategy to precisely manipulate geometric shapes, cell‐cell interactions, ECM composition, dynamics, and presence of crucial soluble factors at the macroscopic scale, thereby enabling the reconstruction of intricately patterned intestinal organoids (**Figure** **8**a).^[^ ^130^ ^]^ Upon the introduction of specific growth factor mixtures, murine ISCs formed organoids that resembled the epithelial structure of the native small intestine, featuring crypts and villus‐like compartments, and which further developed into epithelial tubes. Guided by cylindrical geometries, these bioprinted organoids condensed into thick tubular structures devoid of lumens, subsequently expanded into colonies, and eventually merged into polarized and luminalized epithelial tissues, which began budding 4–6 days later. This complex organoid formation resulted from cellular reshaping and self‐organizing processes following bioprinting, which reduced printing time and geometric complexity, and enhanced functionality and similarity to normal tissues. Combining ISCs with bioprinting to create organoids provides robust tools for engineering self‐organizing tissues, simulating organ boundaries, and exploring tissue‐tissue interactions.

Ren et al. utilized decellularized ECM from the submucosa of porcine small intestines as bioink, which offered various advantages, including biological activity, biocompatibility, tunable stiffness, and excellent printability (Figure 8b).^[^ ^131^ ^]^ This bioink was combined with ISCs to bioprint intestinal organoids, which provided specific ECM content and biomimetic microstructures. This approach demonstrated a robust capacity for organoid formation, as well as unique differentiation patterns and transcriptional features. These bioprinted organoids not only served as an ideal model for studying epithelial homeostasis and interactions, but also promoted organoid maturation upon transplantation into the mesentery of immune‐deficient mice.

Nancy and colleagues isolated intestinal crypts from human tissue and then cultured self‐renewing human intestinal organoids on a collagen scaffold (Figure 8c).^[^ ^132^ ^]^ Growth factors, cytokines, and bacterial metabolites were placed in the reservoir beneath the bioprinted intestinal organoids to investigate the effects of the gradient structure on crypt polarization in the organoids, including stem cell proliferation and the proportions of different cell types at crypt sites. In vitro experimental results showed that the bioprinted intestinal organoids accurately mimicked luminal accessibility, in vivo crypt structure, tissue polarity, cell migration, and cellular responses, making them an ideal model for studying dynamic intestinal behaviors.

The construction of intestinal tissues in vitro posed challenges due to their role in absorption and secretion, requiring the support of various tissues such as lymphatic, vascular, smooth muscle, and neural components.^[^ ^133^ ^]^ An ideal intestinal organoid should replicate the essential functions of native tissues, ensuring lumen patency and optimal mechanical properties while avoiding immune responses.^[^ ^134^ ^]^ The fabrication of intestinal organoids through bioprinting is pivotal for enhancing cell survival, integration, and overall functionality.^[^ ^135^ ^]^ Additionally, addressing the challenge of improving neural innervation via intestinal neurons is crucial for enhancing the contractility, secretion, and motility of these bioprinted organoids.

In addition to the above ASCs, other types of stem cells also played pivotal roles in constructing organoids and treating diseases, including adipose‐derived stem cells and hematopoietic stem cells.^[^ ^136^ ^]^ The utilization of ASCs was more advantageous in specific contexts compared with iPSCs. ASCs were more readily available in larger quantities and had the ability to proliferate, and could differentiate into desired cell types. At the same time, ASCs provided a solution to ethical and legal concerns associated with the use of human oocytes, embryos, and fetuses as sources of stem cells, and addressed issues related to mutations and other adverse effects associated with iPSCs.^[^ ^137^ ^]^ When regenerative therapy was required, ASCs could be isolated and applied in an autologous manner, derived from the patient's own extra‐fetal tissues.^[^ ^138^ ^]^ However, it was worth noting that this approach tended to be more costly, and had limited storage capacity, and resulted in diminished cell viability over time.

## 5 Bioprinted Organoids Derived from Cancer Cells

Compared to traditional methods where cells spontaneously differentiate and aggregate to form tumor organoids, bioprinting uses bioinks containing cells, ECM, and growth factors to construct a structured tumor model layer by layer. Bioprinted cancer organoids have demonstrated significant potential, propelling traditional 2D cancer cell cultures into more intricate 3D cancer models in vitro.^[^ ^139^ ^]^ These cancer organoids have emerged as a prominent platform for cancer pathology research and translational applications, and held crucial value in summarizing pathology, genetic, and phenotypic characteristics of patient‐derived tumor tissues.^[^ ^140^ ^]^ Compared to traditional cell culture, cancer organoids more accurately portrayed features of the original cancer and replicated cancer microenvironment‐cell interactions through co‐culturing with non‐cancer cells. Cancer organoids have been used in precision medicine through high‐throughput screening platforms to assess the sensitivity of anticancer drugs.^[^ ^141^ ^]^

### 5.1 Organoids Derived from Breast Cancer Cells

Breast cancer stood is the most prevalent and diverse malignant tumor affecting women on a global scale, giving rise to significant health challenges.^[^ ^142^ ^]^ Unfortunately, due to species disparities and variations in tissue structure, both in vivo models and traditional cell culture techniques for constructing breast cancer models frequently encountered higher failure rates. To address this pressing issue, the cultivation of breast cancer cells derived from patients into organoids has emerged as a highly promising alternative.^[^ ^143^ ^]^ These organoids faithfully retained the original cancer's diversity, facilitating the expansion of cancer samples and representing distinct stages of cancer development.^[^ ^144^ ^]^ When cultured under precise conditions, these bioprinted organoids offer many advantages, including convenient sample collection, reduced cultivation time, and genetic stability.^[^ ^145^ ^]^

**Figure 9:**
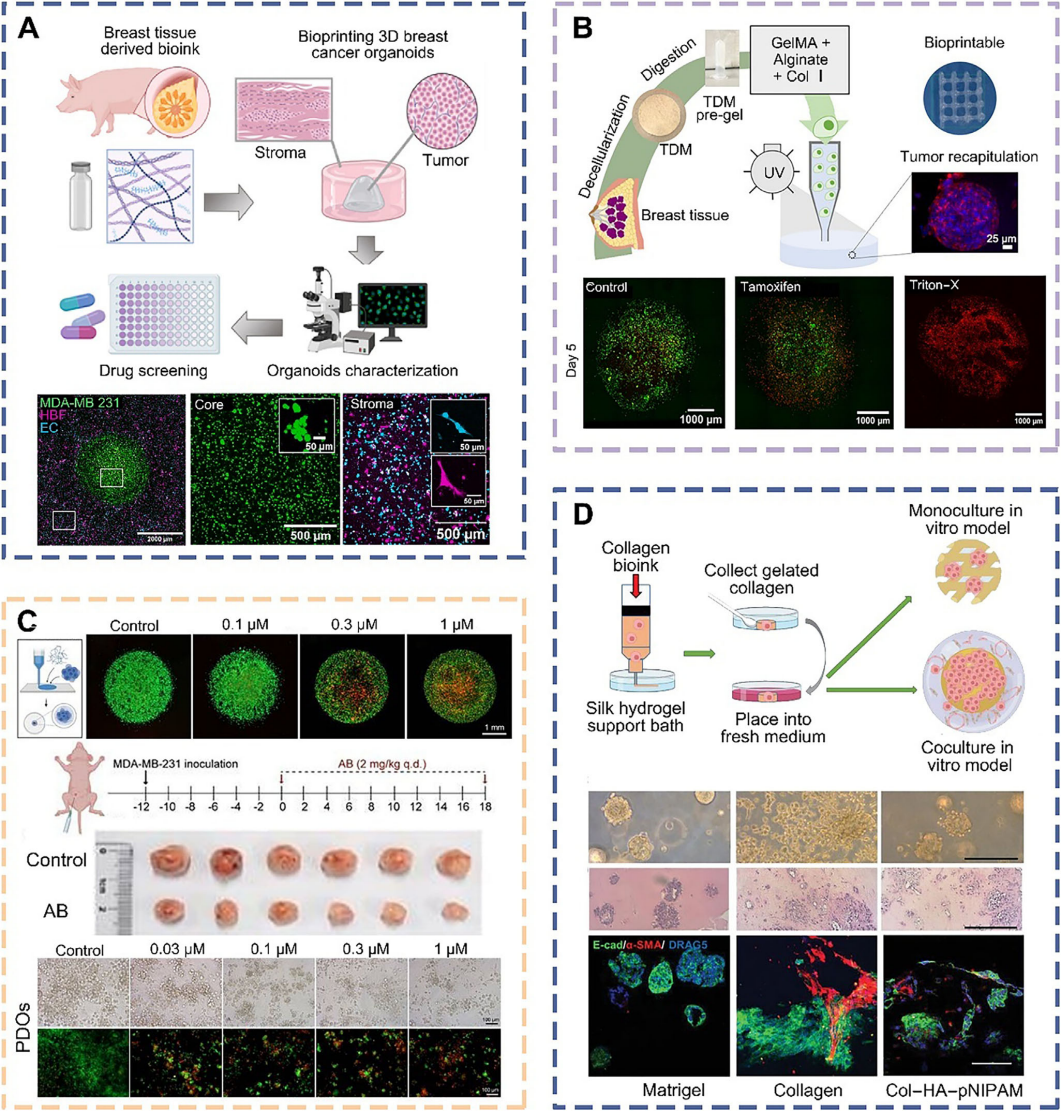
Schematic diagrams and applications of bioprinted organoids derived from breast cancer cells. a) Schematic diagrams of bioprinted breast tumor‐stroma organoids for high‐throughput drug screening, along with live‐cell confocal fluorescence images.^[^ ^146^ ^]^ Copyright 2023, Elsevier. b) Schematic diagrams of bioprinted cancer organoids and confocal fluorescence images from live/dead viability assays.^[^ ^147^ ^]^ Copyright 2022, ACS. c) Schematic diagrams of bioprinted breast cancer organoids and in vivo drug validation results.^[^ ^149^ ^]^ Copyright 2016, ACS. d) Schematic diagrams of bioprinted breast cancer organoids using collagen‐based bioink.^[^ ^150^ ^]^ Copyright 2023, Wiley.

Luis and colleagues combined decellularized ECM with methacrylated hyaluronic acid as bioink for bioprinted breast cancer organoids derived from triple‐negative breast cancer cells (**Figure** **9**a).^[^ ^146^ ^]^ These organoids accurately replicated the structural and biological characteristics of the breast cancer microenvironment and captured the diversity of cell populations and pathways promoting cancer growth. Moreover, this design allowed for the study of biological signaling pathways involved in cancer progression, serving as an ideal model for high‐throughput drug screening. Through the bioprinted breast cancer organoids and the integration of a diverse range of biomolecular characterizations, immune cells, and patient‐derived cancer samples, it became feasible to more precisely represent the cancer microenvironment, interactions with the immune system, and individual patient responses to treatment. This approach held significant importance in expediting drug discovery, refining treatment strategies, and enhancing cancer research and clinical practices.

Alginates and GelMA were blended into porcine mammary ECM to create hydrogels with appropriate firmness, and these hydrogels were then combined with cancerous breast cells as bioink for bioprinted breast cancer organoids in vitro (Figure 9b).^[^ ^147^ ^]^ The resulting organoids exhibited increased viability and proliferation of MCF‐7 cells, facilitating the formation of cell clusters and spheroids with reduced E‐cadherin expression, elevated tumor marker expression, and decreased responsiveness to chemotherapy. These results underscored the substantial potential of bioprinted breast cancer organoids, not only as models for studying cancer progression, but also as platforms for in vitro drug research.

Luan et al. constructed a human breast cancer organoid by bioprinting MDA‐MB‐231 cell spheroids and used it to evaluate the efficacy of anticancer drugs (Figure 9c).^[^ ^148^ ^]^ The experimental results demonstrated that the bioprinted breast cancer organoid exhibited the same response as patient‐derived organoids, and both models confirmed the antitumor effects of Aurovertin B (AB). This study showed the potential of bioprinted organoids as highly effective in vitro tumor diagnostic models, which could significantly aid in identifying patient‐specific therapeutic drugs.

Duan et al. employed a low concentration of collagen and thermoresponsive hyaluronic acid as bioink, with silk fibroin hydrogels providing support, to bioprint breast cancer organoids (Figure 9d).^[^ ^150^ ^]^ This bioprinted cancer organoids exhibited a complex structure incorporating at least three types of cells, preserving morphologies similar to those of primary breast cancer. Additionally, they developed vascularized cancer organoids and noted an increase in VEGF secretion by cancer cells, significantly enhancing the formation of vascular system under hypoxic conditions. These findings suggested that bioprinted breast cancer organoids offered a promising platform for studying the cancer microenvironment and personalized drug screening.

Bioprinted organoids derived from breast cancer cells from a patient served as a cornerstone for fundamental research and clinical application, including cancer modeling, investigation of cancer development mechanisms, and drug screening.^[^ ^151^ ^]^ However, these organoids lacked vascularization processes and failed to replicate the complete microenvironment, including neovasculature, stroma, and immune cells, thus impeding further clinical translation. Therefore, the development of mature bioprinted breast cancer organoid to provide customized treatment for patients represents a critical area for future research in organoid filed.^[^ ^152^ ^]^

### 5.2 Organoids Derived from Lung Cancer Cells

As a malignant tumor, lung cancer ranks as a leading cause of cancer‐related mortality worldwide.^[^ ^153^ ^]^ The primary risks associated with lung cancer include high rates of metastasis and recurrence, for which effective clinical treatments remain lacking. Therefore, the development of potent anti‐cancer drugs represents a crucial pathway for advancing cancer therapy. Traditional methods involved 2D models of cancer cells in monolayer cultures to explore tumor initiation and progression, as well as to evaluate the efficacy of anti‐cancer medications.^[^ ^154^ ^]^ However, 2D models often lose or alter certain original features and functions of normal cancer tissues, such as cell‐cell and cell‐matrix interactions.^[^ ^155^ ^]^ Thus, the development of bioprinted lung tumor organoids that are more reliable, effective, and capable of simulating the actual in vivo environment is essential for understanding of cancer initiation and progression, as well as for screening anti‐cancer drugs.^[^ ^156^ ^]^

**Figure 10:**
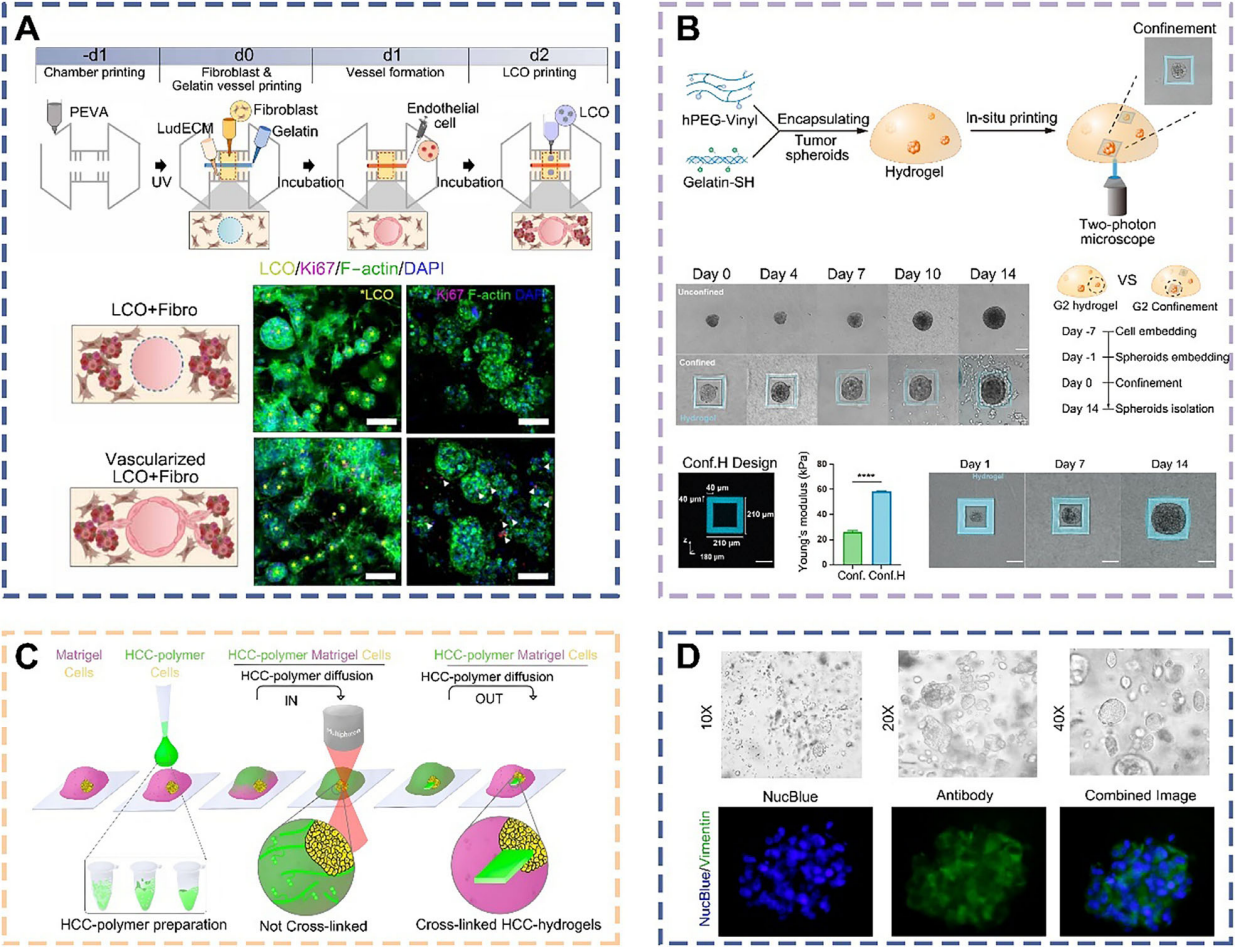
Schematic diagrams and applications of bioprinted organoids derived from lung cancer cells. a) Schematic diagrams of bioprinted vascularized lung cancer organoids and direct interaction between HUVECs and fibroblasts in organoids. Scale bar, 500 µm.^[^ ^157^ ^]^ Copyright 2023, IOP. b) Schematic diagrams of bioprinted lung cancer organoids in vitro and their effects on regulating cell progression.^[^ ^158^ ^]^ Copyright 2025, Elsevier. c) Strategy and set‐up for hydrogel‐in‐hydrogel live bioprinted organoids.^[^ ^159^ ^]^ Copyright 2023, Springer Nature. d) Representative bright‐field and immunostaining images of cell spheroids within the bioprinted lung cancer organoids.^[^ ^160^ ^]^ Copyright 2019, Springer Nature.

In an effort to comprehensively characterize the biochemical composition of natural lung tissue, Jang et al. utilized decellularized ECM hydrogels extracted from pig lungs as bioink to bioprint organoids (**Figure** **10**a).^[^ ^157^ ^]^ These advanced vascularized lung cancer organoids demonstrated enhanced proliferative activity compared to non‐vascularized lung cancer organoids. This vascularized lung cancer organoid replicated the process of lung fibrosis, enabling assessment of drug responsiveness and facilitating the determination of suitable personalized treatment plans for patients.

Nicola et al. developed a tunable bioink based on PEG and gelatin, allowing precise control over density and stiffness, which enabled the bioprinting of multicellular lung cancer spheroids to generate organoids (Figure 10b).^[^ ^158^ ^]^ By utilizing micron‐scale structural constraints, they investigated the impact of the tumor microenvironment on cell cycle‐related pathways. The experimental results revealed that the ECM modulates localized confinement stress through distinct mechanotransduction mechanisms, thereby regulating cancer cell progression. This study demonstrates the value of bioprinted lung cancer organoids, combined with spatial transcriptomics, localized force measurements, and ultra‐high‐resolution real‐time imaging, for advancing our understanding of mechanotransduction in lung cancer cells and metastatic processes, providing crucial insights for future cancer research.

Anna et al. developed a bioink based on 7‐hydroxycoumarin‐3‐carboxylic acid (HCCA), which allows dynamic modulation of the spatial architecture and mechanical environment essential for the 3D culture of organoids.^[^ ^159^ ^]^ This system effectively guides and regulates cellular behavior within bioprinted organoids. In their study, organoids derived from A549 lung adenocarcinoma cells were first cultured in vitro, and the impact of bioprinting on organoid growth and cancer cell migration was subsequently assessed. The results indicated that while bioprinting did not affect cell viability, it did restrict organoid morphology and cancer cell migration. These findings demonstrate that bioprinted lung cancer organoids can be fabricated with customizable sizes and geometries, providing a valuable platform for studying cancer cell migration dynamics within diverse tissue architectures.

Mandip et al. utilized a sodium alginate–gelatin hydrogel to bioprint cancer‐associated fibroblasts with patient‐derived xenograft cells from non‐small cell lung cancer, successfully creating an in vitro lung cancer organoid that mimics key features of the tumor microenvironment (Figure 10d).^[^ ^160^ ^]^ This bioprinted organoid exhibited excellent biocompatibility, with both cell types showing high viability and forming 3D co‐cultured spheroids. Functional assays further revealed crosstalk between the cells within the organoid, suggesting its potential utility for high‐throughput drug screening and other preclinical applications.

The bioprinted lung cancer organoids successfully incorporated stromal cells and cancer cells into a 3D structure, effectively emulating the growth process and cellular functionality of natural cancer tissue, thereby creating an ideal research model.^[^ ^161^ ^]^ These organoids played a pivotal role in pathology and biology research, facilitated drug screening, and served as a crucial preclinical testing tool for addressing diverse medical research issues, such as functional organ replacement, regenerative medicine, and drug delivery. Nevertheless, bioprinted lung cancer organoids necessitated the consideration of additional constraints, encompassing a more intricate cancer microenvironment and the characteristic spatial distribution of cell behavior, in order to more accurately construct lung cancer organoids in vitro.^[^ ^162^ ^]^

### 5.3 Organoids Derived from Liver Cancer Cells

In the 2021 statistics from the World Health Organization, liver cancer emerged as a significant global health issue, with approximately one million individuals diagnosed worldwide each year.^[^ ^163^ ^]^ As a result, the development of a robust liver cancer organoid was essential for research into the origin and pathophysiology of this disease.^[^ ^164^ ^]^ A durable liver cancer organoid, which replicated the diseased state of liver cancers in vitro, could serve to assess drug dosage toxicity, improve the efficiency of preclinical testing, and forecast clinical outcomes. Harnessing the benefits of bioprinted organoids derived from liver cancer cells in vitro was vital for undertaking extensive carcinogenic studies and offering personalized treatment options.^[^ ^165^ ^]^

**Figure 11:**
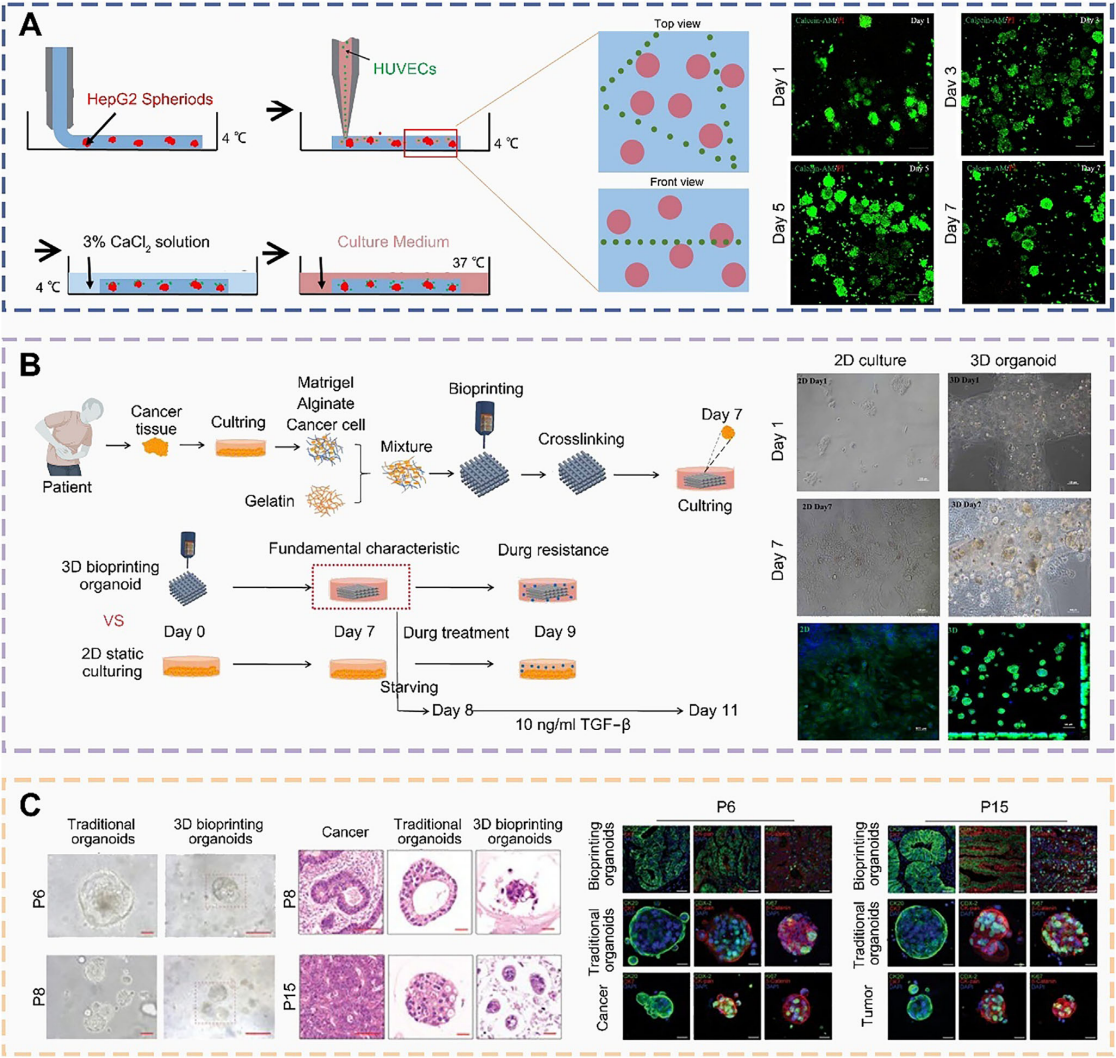
Schematic diagrams and applications of bioprinted organoids derived from liver cancer cells. a) Schematic diagrams of bioprinted liver cancer organoids showing the simultaneous patterning of HepG2 spheroids and labeled HUVECs. Scale bars, 200 µm.^[^ ^166^ ^]^ Copyright 2019, Elsevier. b) Schematic diagrams of bioprinted organoids derived from liver cancer cells, along with the evaluation of resistance evaluation. Scale bars, 100 µm.^[^ ^167^ ^]^ Copyright 2020, IOP. c) Bright‐field, HE, and fluorescence images of bioprinted organoids derived from colorectal cancer liver metastasis. Scale bar, 100 µm (cancer tissue) and 20 µm (bioprinted and traditional organoids).^[^ ^168^ ^]^ Copyright 2023, Wiley.

Sun et al. integrated extrusion‐based bioprinting with alternating viscoelastic and inertial force jetting technology by switching dual nozzles, successfully creating a heterogeneous liver cancer organoid containing HepG2 cell spheroids and HUVECs (**Figure** **11**a).^[^ ^166^ ^]^ The bioprinted liver cancer organoid exhibited excellent cell viability, sustained cell growth, and enhanced cellular functions, facilitating interactions between single cells and spheroids. These findings suggest that bioprinted liver cancer organoids can better mimic cell arrangements and reconstruct the liver cancer microenvironment to support cellular functions. The in vitro reconstruction of liver cancer models provides valuable insights for drug development and disease progression studies.

Sun et al. utilized a composite hydrogel consisting of collagen, alginate, and Matrigel as bioink, which was mixed with intrahepatic cholangiocarcinoma cells isolated from patients to bioprint liver cancer organoids (Figure 11b).^[^ ^167^ ^]^ The cholangiocarcinoma cells in organoids displayed high viability, active proliferative colony‐forming ability, and potential for tissue invasion and metastatic phenotypes. These results implied that bioprinted organoids were capable of replicating the growth process of liver cancer in vivo, positioning them as an exemplary model for studying cancer pathology and development. The observed drug resistance of the cells within the organoids further confirmed their tumor‐like characteristics, highlighting the potential of bioprinted liver cancer organoids for personalized treatment, particularly in targeted drug screening.

Mao et al. created a bioink by uniformly blending gelatin and sodium alginate with a suspension of tumor cells obtained from patients (Figure 11c).^[^ ^168^ ^]^ They utilized a bioprinting strategy to generate grid‐like liver cancer organoids, which showcased specific biomarkers and characteristic mutation spectra consistent with their parental tumors. Drug testing revealed heterogeneity among organoids from different patients, as well as between organoids derived from primary and metastatic tumor cells from the same patient. The construction of tumor organoids from patient‐derived liver cancer cells ex vivo allows for personalized drug response screening and correlates with clinical outcomes following adjuvant therapy in patients. This further solidifies the reliability and efficacy of this platform for cancer treatment.

The development of bioprinted organoids, in combination with specific types of liver cancer cells, has shown progress from theoretical applications to practical utility. This advancement holds the promise of offering assistance in clinical treatments in the short‐term. The advantages of bioprinted organoids, with favorable biocompatibility and stability, enable the replication of the architecture of liver cancers, serving as personalized and customized research models.^[^ ^169^ ^]^ Whether for investigating the pathophysiological processes of cancers or screening specific drug, these models could fulfill roles that conventional methods struggle to achieve.^[^ ^170^ ^]^ However, it is essential to acknowledge that bioprinted liver cancer organoids are still at the experimental stage and require further research to ensure their safety and efficacy before proceeding to subsequent clinical validation. This transformative technology has increasingly garnered attention and holds the potential to provide an optimal platform for personalized medicine, preclinical diagnostics, drug screening, and pathological research.

Currently, bioprinted organoids are primarily focused on studying cancer occurrence, potential molecular mechanisms, drug screening, and treatment strategies.^[^ ^171^ ^]^ However, it still presentes certain limitations, such as the absence of a complete microenvironment that includes neural, vascular, and immune cells, which are crucial for faithfully replicating the natural cancer tissues. This limitation results in cancer organoids failing to represent all cancer characteristics, thereby altering the morphology and function of cells in organoids and ultimately leading to the loss of tissue heterogeneity.^[^ ^172^ ^]^ Heterogeneity is a critical indicator of the biological characteristics of a cancers and significant area of study for drug resistance, recurrence, and metastasis, impacting the efficacy of cancer treatment and contributing to lower reproducibility of bioprinted organoids in vitro research results in clinical trials.^[^ ^173^ ^]^ Therefore, the development of bioprinted cancer organoids will concentrate on various aspects, including enhancing generation efficiency, shortening cultivation periods, standardizing preparation protocols, regulating culture conditions, and reducing cultivation costs, all with the aim of expediting clinical application.

## 6 Summary and Outlook

Due to intricate microenvironments and cellular distribution, the fabrication of human tissues and organs by traditional cell biology techniques presents significant challenges. Bioprinted organoid have already yielded a multitude of promising studies, they demonstrate the capacity to fabricate in vitro models with enhanced reliability, precision, and high‐throughput.^[^ ^174^ ^]^ Organoids are particularly advantageous because they biomimic real‐life physiological conditions in vitro, thereby minimizing reliance on animal models and decreasing inherent variability between animal models and humans. Consequently, bioprinted organoids show significant advancement due to their ability to create physiologically relevant models through bioinspired approaches. Different cell sources for bioprinted organoids indicates unique advantages. Based on the above analysis, their advantages, disadvantages, and application areas are systematically summarized in **Table** **3**.

**Table 3:** The advantages, disadvantages, and application of different cell sources for bioprinted organoids.

Cell sources	Advantages	Disadvantages	Applications
ESCs	Pluripotency High proliferation Early modeling	Ethical issues Tumor risk Immune rejection	Embryogenesis genetic disease models
iPSCs	Ethical compliance Patient‐specificity Gene editing	Reprogramming Epigenetic memory High cost	Personalized therapy Cancer research
ASCs	Tissue specificity Low tumor risk Rapid generation	Limited differentiation Poor scalability Donor dependency	Tissue repair Infection/ drug testing
Cancer cells	Tumor heterogeneity Personalized testing Microenvironment	High failure rates Genetic instability Limited representation	Precision oncology Drug screening Metastasis studies

The combination of bioprinting and cellular technologies has proved the potential to fabricate organoids, paving the way for a new epoch in the fields of biotechnology and medicine. In contrast to conventional approaches, bioprinted organoids present several distinct advantages:

- **Stability**: By implementing standardized processes, material formulations, and precise printing parameters, bioprinted organoids achieve uniform structure and function, thus improving the reliability and reproducibility essential for clinical and research applications.^[^ ^175^ ^]^
- **Efficiency**: Bioprinted organoids allows for meticulous control over cell distribution and biomaterial utilization, thereby expediting cellular activity, optimizing resource usage, and significantly improving the efficiency of in vitro organoid construction.^[^ ^176^ ^]^
- **Versatility**: By leveraging computer‐aided design capabilities, bioprinted organoids can precisely fabricate organoids with intricate and controlled interactions between cells and ECM, effectively replicating the complex biological functions of natural tissues and organs.^[^ ^177^ ^]^
- **Personalization**: Bioprinted organoids cater to the individualized needs and nuances of each patient, offering highly specific and compatible models based on the patient's physiological characteristic, providing valuable insights into disease‐specific progression.^[^ ^178^ ^]^

While bioprinted organoids have made impressive strides, there are ongoing challenges that must be overcome. Extending the longevity of bioprinted organoids presents critical challenge, as it is essential for the development of fully functional, mature tissues that achieve stable equilibrium. Vascularization is critical for replicating the physiological conditions necessary for organoid function, while the formation of neural networks is essential for enabling organoid functionality and simulating the complex neural regulation of organs. It is necessary to improve capacity for high‐fidelity resolution and reconstruction of vascular networks of bioprinted organoids, which are essential for sustaining cell vitality over extended periods and achieving precise control over cellular density.^[^ ^179^ ^]^ Future research must develop the more efficient vascularization strategies, particularly through the optimization of biomaterials and cellular sources to support the formation of intricate and stable vascular networks^[^ ^180^ ^]^ Simultaneously, efforts should focus on precise cell arrangement, synapse formation, and simulating electrical activity to build more complex neural networks. Ultimately, the co‐formation of neural and vascular networks lays the foundation for generating functional organoids and physiologically relevant in vitro models through bioprinting.

Moreover, the selection of materials for bioprinted organoid is currently constrained, as only a subset of materials is compatible with the physiological temperature range of the human body, and certain materials may negatively impact cells.^[^ ^181^ ^]^ However, despite its widely adopted for organoid culture because of its ability to mimic a physiologically relevant microenvironment, Matrigel has several limitations, including batch‐to‐batch variability and challenges in precisely controlling its composition. Future bioprinting inks must be developed to overcome these challenges in order to achieve sufficient raw materials for bioprinted organoids.^[^ ^182^ ^]^ Hydrogels such as alginate, gelatin, and fibrin have been proposed as potential alternatives, but they often fall short in providing the structural support and cell‐matrix interactions needed under the sensitive conditions of organoid culture. Ideally, bioinks should offer a balance of biocompatibility, structural integrity, and tunability, allowing for precise control over cell organization, tissue architecture, and the microenvironment. Successful development of ideal bioinks is crucial for the successful application of bioprinted organoids in regenerative medicine.

At the same time, the existing methods for bioink preparation and the bioprinting process need substantial improvement.^[^ ^183^ ^]^ Machine learning can be leveraged as a powerful tool for this purpose, for example, to manage cell quality, refine printing parameters, or forecast the cell count in individual bioink droplets.^[^ ^184^ ^]^ The integration of six‐axis design principles into bioprinting has opened new avenues for printing cell in any desired orientation within a 3D framework.^[^ ^185^ ^]^ This innovation facilitates the creation of cardiac tissues replete with capillary networks, effectively overcoming the traditional challenge of poor integration between cells and vascular networks in layer‐by‐layer printing. Additionally, investigating the genesis and maturation of organs under diverse environmental conditions presents a formidable research frontier, such as the development of bioprinted organoids in the space environment.^[^ ^186^ ^]^ With the dispatch of the inaugural bioprinter to the International Space Station, research into bioprinted structures under microgravity has already begun, heralding a new era of exploration in this domain.^[^ ^187^ ^]^

The development of bioprinted organoids is not only inseparable from breakthroughs in biomaterials and the creation of suitable environments, but also need the integration of other cutting‐edge technologies, accelerating their progress and application. For instance, leveraging gene editing technologies allows for the simulation of various genetic mutations within bioprinted organoids, thereby creating more realistic disease models and significantly enhancing the speed and success rate of drug development.^[^ ^188^ ^]^ Meanwhile, artificial intelligence can optimize the bioprinting process through data analysis, pattern recognition, and predictive modeling.^[^ ^189^ ^]^ It can also handle large‐scale data processing to uncover crucial insights into cellular behavior and biological interactions within the organoids. The integration of these advanced technologies is expected to enable bioprinted organoids to deliver more efficient and personalized treatment solutions, providing stronger support for organ transplantation and drug development.

In addition to the above contents, but the associated risks of bioprinted organoids must also be carefully considered. One important issue is the ethical and legal controversies surrounding the use of embryonic or stem cells during the bioprinting process.^[^ ^190^ ^]^ Robust regulatory frameworks are required to ensure that these practices align with contemporary scientific and ethical standards. Another concern is the impact of bioprinting on social equity and access to technology. Bioprinted organoids could exacerbate inequalities in the global distribution of healthcare resources. It is crucial to ensure that the widespread adoption of this technology does not lead to future social disparity. Additionally, issues such as data privacy, data misuse, and the intended use of such technologies raise important questions, which demand stricter societal oversight to ensure that bioprinted organoids contribute to medical progress without introducing ethical risks.^[^ ^191^ ^]^

While the transition of bioprinted organoids from laboratory innovation to clinical practice still presents certain challenges, advancements in drug discovery, personalized medicine, and gene therapy underscore the vast potential of this technology. Bioprinted organoids have the capacity to replicate the intricate structures and functions of human organs, providing a novel methodology for delving into complex biological questions. These developments are forging new pathways in biomedical research and clinical applications. There is optimistic expectation that bioprinted organoids will achieve further breakthroughs across a broader range of fields and gain wider acceptance in the future.

## Conflict of Interest

The authors declare no conflict of interest.

## Author Contributions

Z.L. wrote the original draft, conceptualized the study, and acquired funding. C.Z., K.L., Y.Z., Y.G., J.H., S.C., X.F., and K.L. reviewed and edited the manuscript. P.Z., Z.C., and C.R. supervised the work, reviewed and edited the manuscript, and conducted the investigation.
